# Susceptibility to false discovery in biomarker research using liquid chromatography–high resolution mass spectrometry based untargeted metabolomics profiling

**DOI:** 10.1002/ctm2.469

**Published:** 2021-06-27

**Authors:** Pengwei Zhang, Irene L. Ang, Melody M.T. Lam, Rui Wei, Kate M.K. Lei, Xingwang Zhou, Henry H.N. Lam, Qing‐Yu He, Terence C.W. Poon

**Affiliations:** ^1^ The First Affiliated Hospital & MOE Key Laboratory of Tumor Molecular Biology Jinan University Guangzhou China; ^2^ Pilot Laboratory Institute of Translational Medicine Centre for Precision Medicine Research and Training Faculty of Health Sciences University of Macau Macau China; ^3^ Proteomics Core Institute of Translational Medicine Faculty of Health Sciences University of Macau Macau China; ^4^ Department of Biochemistry and Molecular Biology Zhongshan School of Medicine Sun Yat‐Sen University Guangzhou China; ^5^ Department of Chemical and Biological Engineering Hong Kong University of Science and Technology Hong Kong China

Dear Editor,

Our study demonstrates that biomarker research using liquid chromatography (LC)‐high resolution (HR) mass spectrometry (MS) based untargeted metabolomics profiling is susceptible to the discovery of false positive biomarkers.

LC‐MS, especially LC‐HRMS, is popularly used to discover putative biomarkers through comparing untargeted metabolomic profiles between a patient group and a control group.^1^, ^2^ This approach is susceptible to various pre‐analytical, analytical, and post‐analytical biases.^3^ Moreover, isotopes, adducts, in‐source fragment products of some metabolites, artifacts, and contaminants could be wrongly considered as unique metabolomic features.^4^, ^5^ To what extent the putative metabolomic biomarkers could be false remains unknown.

We attempted to identify putative biomarkers for differentiating two artificial groups of plasma samples (12 samples in each group) with well‐defined differences in their metabolome contents (Figure 1A, Table 1; Tables S1 and S2). By design, a maximum of 22 putative biomarkers are true, and the rest of the putative biomarkers must be false.

**FIGURE 1:**
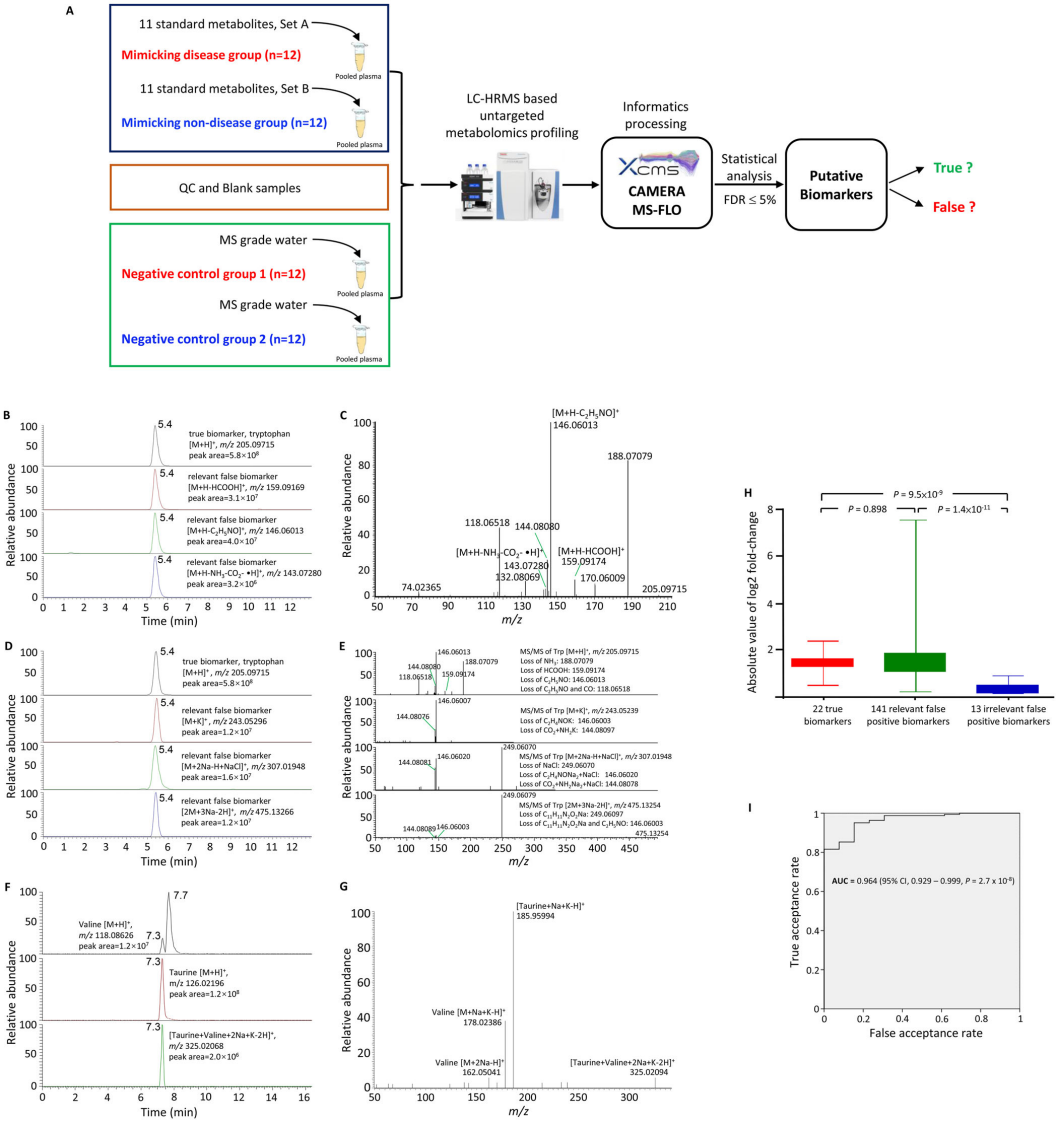
The experimental workflow of the present study (A), MS and MS/MS spectra of representative relevant false positive biomarkers (B – G), and the observed change‐folds of true biomarkers and false positive biomarkers (H, I). (A) When using real patient samples for biomarker discovery, it is not possible to tell which putative biomarkers are false, and it is also not possible to avoid pre‐analytical biases. To overcome these two problems, two groups of plasma samples (12 samples in each group) mimicking those collected from a disease group and a non‐disease group were created by spiking separately with two different sets of 11 metabolite standards into a preparation of pooled human plasma. Each of the 24 plasma samples (i.e., 12 from the disease group and 12 from the non‐disease group) was treated as a unique patient sample and subjected to separate metabolite extraction and LC‐HRMS based untargeted metabolomics profiling. XCMS was employed for feature extraction, grouping and retention time alignment. CAMERA and MS‐FLO were used to annotate and remove the redundant peaks. At a false discovery rate of ≤ 5%, metabolomic features with statistically significant differential intensities were regarded as putative biomarkers. By the design, a maximum of 22 putative biomarkers could be true, and the rest of the putative biomarkers were false. Six relevant false positive biomarkers which were identified as the in‐source fragmentation products (B, C) and adducts (D,E) of a true biomarker (e.g., Tryptophan, Trp). The retention times of the in‐source fragmentation products (B) and adducts (D) was the same as that of the original metabolite. The *m/z* values of the in‐source fragmentation products (B) identical to the *m/z* value of one of the peaks in the MS/MS spectrum of the original metabolite (C). The MS/MS fragmentation patterns of the adducts (D) and the principal ion of original metabolite shared common fragment ions (E). An example of a relevant false positive biomarker which was identified as in‐source complex of a true biomarker (Valine) and an endogenous metabolite (Taurine) (F). The extracted ion chromatogram (10 ppm tolerance) of valine (upper panel), taurine (middle panel) and in‐source complex of valine and taurine (lower panel) (F), and the MS/MS fragmentation spectra showed fragments of the relevant false positive biomarker [Taurine+Valine+2Na+H‐2H]^+^ (G). The box‐plots (H) of the absolute log2 fold‐change values of the true biomarkers, relevant false positive biomarkers and irrelevant false positive biomarkers and their comparisons (Mann Whitney test, two‐tailed *P*‐values). ROC curve (I) for differentiation the true biomarkers and relevant false positive biomarkers from the irrelevant false positive biomarkers using various cutoffs of the absolute value of log_2_ fold‐change. For each cutoff, the true acceptance rate was the proportion of true biomarkers and relevant false positive biomarkers having their absolute values of log2 fold‐change higher than the cutoff, whereas the false acceptance rate was the proportion of irrelevant false positive biomarkers having their values higher than the cutoff

**TABLE 1:** Information of the two sets of metabolite standards spiked into the human pooled plasma

Metabolite standard	Set	Reported concentration in human plasma (μM)	Estimated mean concentration (μM) in human plasma	Spiked amount (μM)	Supplemental References
L‐Leucine	A	123 ± 25, 140, 66 to 170	125	250	[17–19]
Creatine	A	44 ± 28, 22.5 to 62	55	110	[20, 21]
L‐Histidine	A	82 ± 10, 78.8 ± 18.9	80	160	[17, 22]
L‐Phenylalanine	A	57 ± 9, 70 ± 10.98, 63.0 ± 11.7	65	130	[17, 23, 24]
D‐(+)‐Glucose	A	4700 to 6100, 5770	5000	10000	[17, 25]
L‐Aspartic Acid	A	54 ± 4, 3 ± 1, 19.3 ± 4.8, 30 ± 14	20	40	[17, 22, 26, 27]
L‐Valine	A	233 ± 43, 212.0 ± 61	230	460	[17, 28]
L‐Alanine	A	333 ± 74	330	660	[17]
L‐Serine	A	114 ± 19, 258 ± 27, 104 ± 26, 159.8 ± 26.6	140	280	[17, 26–28]
L‐Carnitine	A	25.4 to 54.1, 43.6 ± 9.9, 50 ± 30	45	90	[23, 29, 30]
L‐Glutamine	A	586 ± 84, 590	590	1180	[17, 31]
Creatinine	B	35 to 122, 74.12 ± 10.91	75	150	[32, 33]
L‐Proline	B	168 ± 60, 339 ± 51, 163.6	240	480	[17, 26, 34]
Betaine	B	20 to 144, 57.0 ± 15.4	75	150	[17, 35, 36]
L‐Glycine	B	230 ± 52, 255.4 ± 65.9	230	460	[17, 31]
L‐Arginine	B	110 ± 21.4, 99.0 ± 22.8, 82.2 to 140.9	110	220	[23, 37, 38]
L‐Threonine	B	140 ± 33, 127.9 ± 28.2	140	280	[17, 39]
L‐Tryptophan	B	67 to 72, 44 ± 7	50	100	[26, 40]
L‐Lysine	B	188 ± 32, 150 ± 2.1, 183 ± 39	180	360	[17, 41, 42]
L‐Glutamic Acid	B	24 ± 15, 87.0 ± 37.1, 72.0 ± 10.1	85	170	[17, 22, 43]
L‐Asparagine	B	41 ± 10, 40.0	40	80	[17, 44]
Hypoxanthine	B	8.14 ± 2.86, 11.02 ± 3.67	10	20	[45, 46]

The number of metabolomic features depended on the signal‐to‐noise ratio threshold (*snthresh*) used for feature extraction (Table 2). A *snthresh* of 5 had been widely used (Table S3). Using a *snthresh* of 5 and a false discovery rate (FDR) cutoff of 5% for data mining, 22 true biomarkers (i.e., true positives) and 165 false positive biomarkers were observed (Table 3; Table S4). Therefore, the actual FDR (i.e., the false positive rate) was 88% instead of 5% (Table 2). Increasing the *snthresh* and reducing the sample size (e.g., n = 6 for each group) could decrease the number of false positive biomarkers. However, the actual FDR remained > 60% (Table 2; Table S5). We also performed a negative control experiment using two groups of plasma samples (n = 12 for each group) having identical metabolome contents. No differential metabolomic features were found (Supplemental Table S6). This indicated that our experimental methods did not suffer from any obvious pre‐analytical biases and analytical biases.

**TABLE 2:** Summary of the biomarker discovery results (FDR = 5%) obtained by comparing the metabolomic profiles of plasma samples mimicking those collected from diseased subjects (n = 12) and non‐diseased subjects (n = 12)

*snthresh* (signal‐to‐noise ratio threshold) for metabolomic feature extraction	Total number of metabolomic features^a^(M)	Number of differential metabolomic features, i.e., putative biomarkers (PB)	Number of true positives i.e., true biomarkers identified as putative biomarkers (TP)	Number of false negatives (FN = 22 – TP)	Number of false positives, i.e., putative biomarkers which were not the true biomarkers (FP = PB – TP)	Percentage of metabolomic features which were false positives (FP / M)	False Negative Rate for the true biomarkers (FNR = FN / 22)	Actual False Discovery Rate, i.e., False Positive Rate for the putative biomarkers (FPR = FP / PB)
20	402	73	21	1	52	12.9%	4.5%	71.2%
10	578	94	22	0	72	12.5%	0%	76.5%
5	805	187	22	0	165	20.5%	0%	88.2%

^a^ The metabolomic features were cleaned up using CAMERA and MS‐FLO. Features absent in 80% of the samples were discarded. Then features corresponding to the features with CV > 30% in the QC samples were discarded. Moreover, features contributed by the impurities in the metabolomic standards were tracked and excluded from the calculations.

**TABLE 3:** Summary of 22 true biomarkers among the 187 putative biomarkers discovered by comparing the metabolomic profiles of plasma samples mimicking those collected from diseased subjects (n = 12) and non‐diseased subjects (n = 12). The metabolomic features were extracted using a snthresh value = 5

						Normalized signal intensity (mean ± SD)			
Identity	Metabolite standards, Set A or Set B	Retention time	Observed m/z	Theoretical m/z	Mass error (ppm)	Diseased	Non‐diseased	BH adjusted *P*‐value	Fold‐change (Diseased /Non‐diseased)
Hypoxanthine	B	2.8	137.04590	137.04578	0.91	8.9E+07±1.1E+07	1.9E+08±1.1E+07	1.8E‐08	0.5
Creatinine	B	3.4	114.06622	114.06619	0.28	6.7E+08±7.4E+07	1.9E+09±7.4E+07	1.7E‐17	0.4
L‐Tryptophan	B	5.3	205.09714	205.09715	−0.07	3.1E+07±2.0E+06	9.8E+07±2.0E+06	1.6E‐15	0.3
L‐Phenylalanine	A	5.4	166.08625	166.08626	−0.03	1.6E+08±7.0E+06	5.3E+07±7.0E+06	2.3E‐20	3.0
L‐Leucine	A	6.0	132.10191	132.10191	−0.01	1.7E+08±1.5E+07	5.9E+07±1.5E+07	3.3E‐15	2.9
L‐Valine [M+2Na‐H]^+^	A	7.4	162.04997	162.05014	−1.08	1.7E+07±2.0E+06	7.6E+06±2.0E+06	1.2E‐11	2.3
Betaine	B	7.7	118.08623	118.08626	−0.24	3.9E+08±2.9E+07	2.0E+09±2.9E+07	2.7E‐19	0.2
L‐Proline	B	7.8	116.07076	116.07061	1.29	8.7E+07±5.5E+06	3.1E+08±5.5E+06	1.0E‐13	0.3
D‐Glucose [M+Na]^+^	A	7.5	203.05258	203.05261	−0.13	3.8E+07±1.4E+07	1.4E+07±1.4E+07	4.8E‐05	2.7
Carnitine	A	8.5	162.11239	162.11247	−0.52	2.4E+09±2.3E+08	9.1E+08±2.3E+08	1.9E‐14	2.7
L‐Alanine	A	8.6	90.05496	90.05496	0.04	3.7E+07±3.0E+06	1.7E+07±3.0E+06	4.3E‐14	2.2
Creatine	A	8.7	132.07674	132.07675	−0.05	2.7E+08±1.3E+07	1.1E+08±1.3E+07	3.4E‐17	2.4
L‐Threonine	B	8.9	120.06552	120.06552	0.03	5.7E+06±9.1E+05	2.1E+07±9.1E+05	5.7E‐12	0.3
L‐Glycine	B	9.1	76.03930	76.03931	−0.19	3.0E+06±1.4E+05	7.4E+06±1.4E+05	7.4E‐17	0.4
L‐Serine	A	9.4	106.04984	106.04987	−0.25	9.1E+06±1.2E+06	3.4E+06±1.2E+06	1.1E‐11	2.6
L‐Glutamine	A	9.3	147.07636	147.07642	−0.43	6.7E+07±1.2E+07	3.2E+07±1.2E+07	7.1E‐08	2.1
L‐Glutamic acid	B	9.4	148.06034	148.06043	−0.58	2.0E+07±2.4E+06	2.8E+07±2.4E+06	6.4E‐04	0.7
L‐Asparagine	B	9.5	133.06075	133.06077	−0.19	1.3E+06±2.8E+05	5.2E+06±2.8E+05	1.1E‐09	0.2
L‐Aspartic acid [M+2Na‐H]^+^	A	10.0	178.00862	178.00868	−0.30	1.4E+06±5.6E+05	5.1E+05±5.6E+05	5.8E‐04	2.7
L‐Arginine	B	10.4	175.11884	175.11895	−0.62	1.2E+08±1.4E+07	3.8E+08±1.4E+07	1.3E‐16	0.3
L‐Histidine	A	10.4	156.07675	156.07675	0.00	7.6E+07±3.6E+06	2.8E+07±3.6E+06	2.4E‐19	2.7
L‐Lysine	B	10.5	147.11273	147.11281	−0.70	2.4E+07±2.4E+06	7.2E+07±2.4E+06	2.1E‐14	0.3

We revealed identities of 93% (154) of the false positive biomarkers (Table 4; Table S4). Eighty‐five percent (141) of the false positive biomarkers were contributed by in‐source fragmentation products (29), in‐source complexes (23), adducts (79), and isotopes (10) of the true biomarkers (Figure 1B–1G), whereas 8% (13) were contributed by metabolites irrelevant to the true biomarkers (Figure S1). We named these two types of false positive biomarkers as “relevant false positive biomarkers” and “irrelevant false positive biomarkers,” respectively. Those adducts, in‐source complexes, etc. should be considered as false positives because their signal intensities cannot be accurately measured in biological specimens.^8^

**TABLE 4:** Origins of 165 false positive biomarkers obtained using a signal‐to‐noise ratio threshold value of 5

Origins	Classification	Number of false positive biomarkers (number of them having retention times overlapping with those of spiked metabolite standards)
Spiked metabolite standards	In source fragmentation products	29 (29)
	Adducts	79 (79)
	Isotopes	10 (10)
	In‐source complex	23 (23)
	***Subtotal***	***141 (141)***
Irrelevant metabolites	Principal ion	11 (7)
	In source fragmentation products	0 (0)
	Adducts	2 (2)
	Isotopes	0 (0)
	In‐source complex	0 (0)
	***Subtotal***	***13 (9)***
	Unidentified	**11 (9)**
	***Total***	***165 (159)***

Metabolomic features corresponding to 88 (62%) relevant false positive biomarkers were observed in the LC‐HRMS profiles of the respective mixtures of the pure metabolites (i.e., Set A and Set B, Table S7). This confirmed they were originated from the true biomarkers. Although CAMERA and MS‐FLO were used to annotate and remove redundant peaks, redundant features still existed in the final list of metabolomic features (Table S4). Similar annotation mistakes could be found in the previous studies.^6^, ^7^ It was not possible to differentiate the relevant false positive biomarkers from the true biomarkers according to signal intensity change (Figure 1H). However, they could be identified through manual inspection of the MS and MS/MS spectra. A true biomarker and its relevant false positive biomarkers shared nearly the same retention time and common MS/MS fragmentation products (Figures 1B–1G).

For the irrelevant false positive biomarkers, one possible cause for their presence was matrix effect.^9^ In electrospray ionization MS, matrix effect is characterized as an alteration of signal response by co‐eluting substances. Nine (69%) of 13 irrelevant false positive biomarkers had their retention times overlapping with the spiked metabolites (Table 4). Since the negative control experiment did not find any differential metabolomic features, our data suggested that increasing the plasma levels of 22 metabolites (11 in each study group) by about two‐folds were sufficient to alter the “matrix” significantly. Fortunately, fold‐changes of the irrelevant false positive biomarkers were significantly lower than those of the true biomarkers and the relevant false positive biomarkers (Figure 1H). A fold‐change cutoff of 1.5 could reject 11 of 13 irrelevant false positive biomarkers in the present study, thus suggesting that any differential metabolomic features with a fold‐change < 1.5 have a high chance of being false positive.

Unlike real biomarker discovery studies, in the present study “true” biomarkers and other metabolites were identical among the plasma samples within each study group. Such “homogeneous” design allowed us to identify artifacts unambiguously using a small sample size. With respect to the complexity of metabolomes among patient specimens in real life, the false positive rate of putative metabolomic biomarkers in a real study could be higher or lower than the value reported by us. The amount of false positive biomarkers should depend on various factors, such as number, quantity, and properties of true biomarkers, complex interactions between the true biomarkers and background metabolites in individual specimens, detection sensitivity of the MS platform, and the study sample size. We showed that larger sample size (n = 12 vs. n = 6 for each group) resulted in more false positive biomarkers because of higher statistical power. In general, about 50 cases or more for each study group are needed for reliable biomarker discovery.^10^ We speculate that with sufficient statistical power, similar types of false positive biomarkers could be identified.

To our knowledge, this study is the first to demonstrate that the use of LC‐HRMS based metabolomic profiling for metabolite biomarker discovery are susceptible to false discovery. Without knowing the identities, it is risky to treat any statistically differential features as putative biomarkers. Relevant false positive biomarkers could be identified through careful manual inspection of the retention time, MS, and MS/MS information. Informatics tools which can accurately and effectively annotate the LC‐HRMS features need to be developed. Irrelevant false positive biomarkers could be minimized by using an appropriate fold‐change cutoff (Figure 1I). Other approaches, such as multicenter study, validation using an independent patient cohort and orthogonal chemical analysis, should also help eliminate the false positive biomarkers.

## CONFLICT OF INTEREST

The authors declare no conflict of interest.

## AVAILABILITY OF DATA AND MATERIAL

All supplementary tables and figures are provided with the manuscript. The LC‐HRMS raw data files of the present study is available at the NIH Common Fund's National Metabolomics Data Repository (NMDR) website, the Metabolomics Workbench, https://www.metabolomicsworkbench.org, where it has been assigned Project ID PR000757. The data can be accessed directly via it's Project DOI: 10.21228/M8TQ2B.

## Supporting information

SUPPORTING INFORMATION

## References

[1] Pezzatti J , Boccard J , Codesido S , et al. Implementation of liquid chromatography‐high resolution mass spectrometry methods for untargeted metabolomic analyses of biological samples: a tutorial. Anal Chim Acta. 2020;1105:28‐44. 32138924 10.1016/j.aca.2019.12.062

[2] López‐López Á , López‐Gonzálvez Á , Barker‐Tejeda TC , Barbas C . A review of validated biomarkers obtained through metabolomics. Expert Rev Mol Diagn. 2018;18:557‐575. 29808702 10.1080/14737159.2018.1481391

[3] Ju R , Liu X , Zheng F , et al. Removal of false positive features to generate authentic peak table for high‐resolution mass spectrometry‐based metabolomics study. Anal Chim Acta. 2019;1067:79‐87. 31047152 10.1016/j.aca.2019.04.011

[4] Xu YF , Lu W , Rabinowitz JD . Avoiding misannotation of in‐source fragmentation products as cellular metabolites in liquid chromatography‐mass spectrometry‐based metabolomics. Anal Chem. 2015;87:2273‐2281. 25591916 10.1021/ac504118y PMC4354698

[5] Mahieu NG , Patti GJ . Systems‐Level annotation of a metabolomics data set reduces 25000 features to fewer than 1000 unique metabolites. Anal Chem. 2017;89:10397‐10406. 28914531 10.1021/acs.analchem.7b02380 PMC6427824

[6] Senan O , Aguilar‐Mogas A , Navarro M , et al. CliqueMS: a computational tool for annotating in‐source metabolite ions from LC‐MS untargeted metabolomics data based on a coelution similarity network. Bioinformatics. 2019;35:4089‐4097. 30903689 10.1093/bioinformatics/btz207 PMC6792096

[7] Kouřil Š , de Sousa J , Václavík J , Friedecký D , Adam T . CROP: correlation‐based reduction of feature multiplicities in untargeted metabolomic data. Bioinformatics. 2020;36:2941‐2942. 31930393 10.1093/bioinformatics/btaa012

[8] Lu W , Su X , Klein MS , Lewis IA , Fiehn O , Rabinowitz JD . Metabolite measurement: pitfalls to avoid and practices to follow. Annu Rev Biochem. 2017;86:277‐304. 28654323 10.1146/annurev-biochem-061516-044952 PMC5734093

[9] Taylor PJ . Matrix effects: the achilles heel of quantitative high‐performance liquid chromatography–electrospray–tandem mass spectrometry. Clin Biochem. 2005;38:328‐334. 15766734 10.1016/j.clinbiochem.2004.11.007

[10] Xia J , Broadhurst DI , Wilson M , Wishart DS . Translational biomarker discovery in clinical metabolomics: an introductory tutorial. Metabolomics. 2013;9:280‐299. 23543913 10.1007/s11306-012-0482-9 PMC3608878

