# Supplementary material for: Susceptibility to false discovery in biomarker research using liquid chromatography–high resolution mass spectrometry based untargeted metabolomics profiling
Source: Clin Transl Med. 2021 Jun 27;11(6):e469. doi: 10.1002/ctm2.469 (PMC8236120; doi:10.1002/ctm2.469)
Supplement: Supplementary file 1 — SUPPORTING INFORMATION [file CTM2-11-e469-s001.docx]

**Susceptibility to False Discovery in Biomarker Research using LC-HRMS based Untargeted Metabolomics Profiling**

**Pengwei ZHANG, Irene L. ANG, Melody M.T. LAM, Rui WEI, Kate M.K. LEI, Xingwang ZHOU, Henry H.N. LAM, Qing-Yu HE, Terence C.W. POON**

**Supporting Information:**

Supplemental Methods

Supplemental Tables S1 to S7

Supplemental Figure S1

Supplemental References

**Supplemental Methods**

***Materials***

All solvents were of LC-MS grade, and purchased from Thermo (Thermo Scientific Pierce, Rockford, IL, USA). Mass spectrometric grade ammonium formate was obtained from Sigma-Aldrich (St. Louis, MO, USA). Pooled human potassium EDTA plasma was purchased from a commercial company (Equitech-Bio Inc., Kerrville, TX, USA). Twenty-two metabolite standards which are commonly present in human plasma were obtained from commercial companies. Details of the metabolite standards are provided in Supplemental Table S1. Individual metabolites were subjected to LC-HRMS to check for impurities.

***Preparation of plasma samples for the biomarker discovery experiment***

The samples mimicking diseased plasma samples and non-diseased samples (12 samples for each group) were spiked with Set A (diseased group) and Set B (non-diseased group) metabolite standards, respectively. Details of Set A and Set B metabolite standards are provided in Table 1 (see main text). Each set contained 11 metabolite standards. In our preliminary experiments, using a signal-to-noise ratio threshold value of 5 for extracting metabolomic features from the LC-HRMS profiles, about 800 metabolomic features (after removing redundant peaks) were observed in the pooled human samples. The spike of two different sets of 11 metabolite standards into the pooled human plasma to form the diseased and non-diseased samples could mimic a situation that levels of about 2.5% of the predominant metabolomic features (i.e., 22 true biomarkers in total: 11 with increased levels and 11 with decreased levels) were significantly changed in the plasma of the disease subjects. The metabolite standards were selected according to two criteria. First, they have been shown to have clinical significance in previous studies (Supplementary Table S2). Second, their retention times cover various time points of the LC for metabolite separation. The 22 metabolite standards were randomly assigned to the Set A and Set B. Briefly, individual metabolite standard solutions were prepared in LC-MS grade water. Each set of metabolite standards was prepared separately by mixing equal volumes of 11 metabolite standards. The two metabolite mixtures were dried by speed vacuum at room temperature, and redissolved separately in the human pooled plasma to form two master “spiked plasma” samples. The desired amount of each metabolite standard spiked into the pooled plasma was equal to around 2 times of the corresponding plasma concentration estimated from the previously reported values (Table 1 in main text). Since an arbitrary fold-change cutoff of 2 has been commonly used to identify differential features in many metabolomics studies, our spiking approach was aimed to achieve a fold-change of >2 for the spiked metabolites. Each of the two master spiked plasma samples was divided into 12 identical aliquots (each 30 µL), which were treated as plasma samples collected from 12 different people. The spiked plasma samples were stored at -80 ^o^C before use.

***Preparation of the quality control (QC) sample***

The QC sample was prepared by mixing equal volume of the two master “spiked plasma” samples. It was used to check the stability of the system throughout the whole experimental process.

***Preparation of plasma samples for the negative control experiment***

Two groups of negative control samples (12 identical samples for each group) were prepared according to the above strategy, but Set A and Set B metabolite standard mixtures were replaced with LC-MS grade water.

***Preparation of blank sample***

The blank sample was prepared according to the plasma metabolite extraction procedure, but the plasma was replaced with equal volume of MS grade water.[1] The blank sample was used to perform the background subtraction and remove the interfering ions from the experiment process.[1,2] The blank sample was analyzed at both beginning and end of the sequence.

***Plasma metabolite extraction***

Frozen pooled plasma samples were thawed at 4 ^o^C and vortexed for 30 seconds before metabolite extraction. Individual plasma samples were extracted using the methanol extraction method, as previously reported by other research teams.[3,4] All plasma samples were processed in one batch in a random order. Briefly, 30 µL plasma were mixed with 120 µL chilled methanol, and vortexed for 30 seconds. The mixture was incubated at 4 ^o^C for 20 minutes to precipitate the proteins. After incubation, the mixture was centrifuged at 14,000 g for 10 min. Ninety microliters of the supernatant were recovered as the metabolite extract, and stored at -80 ^o^C before LC-HRMS analysis.

***LC-HRMS analysis***

All extracts were subjected to LC-HRMS analysis in a random order. Two microliters of individual metabolite extracts were separated on an amide HILIC (Accucore™ 150 Amide HILIC 2.1×150 mm, Thermo Fisher) column at a flow rate of 0.3 mL/min using a UHPLC system (Dionex UltiMate 3000 RSLC, Thermo Fisher Scientific). The UHPLC system was equipped with a binary pump, an autosampler and a column thermostat. The autosampler was set at 8 °C. The column oven temperature was 30 °C. Mobile phase A was 98% acetonitrile, 2% water and 0.1% formic acid while mobile phase B was 98% water, 2% acetonitrile, 30 mM ammonium formate and 0.1% formic acid. The mobile phase was freshly prepared before use. The LC-HRMS analysis was performed using a quadrupole-orbitrap mass spectrometer (Q Exactive Orbitrap, Thermo Fisher) with an optimized stepwise linear gradient (10% B in the first 2 min, 10% - 30% B in the next 5 min, followed by 30% - 60% in 3 min, and finally 60% - 90% in 5 min). The MS acquisition parameters were as follows: spray voltage, 3.5 kV; capillary temperature, 320 °C; sheath gas flow rate, 45; auxiliary gas flow rate, 25; heater temperature 350 °C; AGC, 3×10^6^; maximum injection time, 200 ms; mass scan range, 50–750; full MS resolution, 70,000 FWHM at m/z 200; spectrum data type, profile. MS/MS spectra were obtained using either “target MS/MS” mode or data-dependent MS/MS mode to obtain the fragmentation patterns of metabolomic features. The MS/MS acquisition parameters were as follows: MS/MS resolution, 17,500 FWHM at m/z 200; AGC, 2×10^5^; maximum injection time, 200 ms; stepped NCE, 20, 40, 50. Standard solutions of Set A and Set B were also analyzed under the same condition for feature identification purpose. The performance of LC-HRMS was assessed by repeat injections (n = 12) of the same metabolome extract from a human pooled plasma sample. Average relative standard deviations (RSDs) of retention time and normalized peak intensity were 0.3% (range: 0.1% to 0.6%) and 3.8% (range: 0.5% to 7.4%), respectively, for 18 representative metabolomic features covering various retention times (2 min to 11 min) and peak intensities (2×10^5^ to 2×10^9^). With respect to the reliable performance, the metabolome extract from each sample was analyzed once by LC-HRMS.

Prior to the analysis sequence start, the LC was run for 1 h at initial gradient, followed by two injections of the solvent blanks and three injections of the metabolite extract of the QC sample to allow column equilibration and conditioning.[5] For quality control, the metabolite extract of the QC sample was injected every five or six plasma samples to monitor the stability of the system during the whole LC-HRMS analysis. Using *snthresh* = 5, 2361 LC-HRMS features were observed for QC samples. The signal intensities of the LC-HRMS features of each QC sample were normalized using median absolute deviation method.[10,11] The median coefficient of variation (CV) of the normalized signal intensities of the LC-HRMS features of the QC samples (n = 9) were 14.2% (interquartile range of 9.2% to 26.9%), suggesting good MS signal stability during the consecutive LC-HRMS runs.

***LC-HRMS data processing***

The program XCMS online was used for feature detection, grouping, automatic integration and retention time correction.[6] Briefly, the background subtracted raw LC-HRMS files (Xcalibur 3.0, Thermo Fisher Scientific) were converted to .mzXML format using the ProteoWizard Msconvert [7] and uploaded to XCMS online. The key parameters of XCMS were set as: polarity, positive; feature detection, matchedFilter; retention time correction, orbiwap; peak alignment, density. The parameter for “fwhm”, “step”, “mzdiff”, “steps” were set at 10, 0.1, 0.005 and 2, respectively.[8] “mzwid”, and “minfrac” were set as 0.025 and 0.5, respectively. Three “snthresh” (signal-to-noise ratio threshold) values (5, 10, 20) were evaluated. Feature grouping and annotation of isotopes and adducts were performed using CAMERA with settings: “sigma” = 6, “perfwhm” = 0.6, “maxcharge” = 3, “maxiso” = 4 and “ppm” = 10.[9] The *m/z* values, retention times and signal response of the metabolomic features were exported.

The molecular features were subjected to the following clean-up steps. First, isotopic peaks, adducts and in-source fragments were annotated using CAMERA and removed from the feature list. Second, molecular features absent in 80% of the samples were removed from further treatment.[12] Third, features in the “diseased” and “non-diseased” samples corresponding to the features with CV >30% in the QC samples were discarded.[13] Fourth, the features with maximum signal response of all samples less than value of 500,000 were also discarded.[2] Finally, the remained features were processed using online tool MS-FLO to identify and remove the redundant peaks.[14] The joint and flagged features by MS-FLO were manually examined in case of misannotation. Only the remaining features were considered as the metabolomic features and subjected to further analysis. The signal intensities of the metabolomic features in each plasma sample were normalized using median absolute deviation method.[10,11]

***Statistical analysis***

The Student's t-test (2 tailed) was used to compare the normalized signal intensities of individual metabolomic features between two study groups. The *P*-values were adjusted with the Benjamini-Hochberg (BH) procedure. Differential metabolomic features with BH adjusted *P*-values <0.05 were regarded as putative biomarkers (FDR <5%). The Mann Whitney test (2 tailed) was used to compare the absolute values of log2 fold-change between two types of putative biomarkers. Receiver operating characteristic (ROC) curve was generated by plotting the true acceptance rate against the false acceptance rate at various cutoffs of absolute values of log2 fold-change of signal intensity. For each cutoff, the true acceptance rate was the proportion of true biomarkers and relevant false positive biomarkers having their absolute values of log2 fold-change higher than the cutoff, whereas the false acceptance rate was the proportion of irrelevant false positive biomarkers having their values higher than the cutoff. All statistical tests were performed using the SPSS (version 24, IBM). In all statistical analyses, metabolomic features contributed by the 6 impurities among the 22 metabolite standards were excluded.

***Identification of metabolomic features***

Differential metabolomic features corresponding to principal ions, in-source fragmentation products, adducts and isotopes of the spiked metabolite standards were identified through comparisons of retention times, *m/z* values (<5 ppm) and MS/MS fragmentation patterns with those of authentic metabolite standards. Differential features corresponding to other plasma metabolites (i.e., metabolites pre-existing in the pooled plasma) were identified by matching MS/MS fragmentation patterns against reference MS/MS fragmentation data in public spectra libraries (NIST and MassBank) using MS-FINDER through the MS-DIAL program (MS^1^ error tolerance 0.005 Da; MS^2^ error tolerance, 0.01 Da).[15,16] For differential features without MS/MS acquisition (due to low intensity), they were annotated with molecular formula by searching the accurate *m/z* against HMDB under 5 ppm error tolerance. The identified differential features were classified into three groups: “true biomarkers” (spiked standards), “relevant false positive biomarkers” (differential features originated from the spiked standards) and “irrelevant false positive biomarkers” (differential features not originated from the spiked standards).

**Supplemental Table S1. Information of the metabolite standards which were spiked into the pooled human plasma sample to mimic the true biomarkers.**

| Index | Metabolite standard | Manufacture | Cat. No. | Grade | Purity |
| --- | --- | --- | --- | --- | --- |
| 1 | L-Leucine | Sigma-Aldrich, USA | PHR1105 | Pharmaceutical Secondary Standard, certified reference material | 99.00% |
| 2 | Creatine | Sigma-Aldrich, SWITZERLAND | C0780 | Anhydrous, batch no. of BCBD4119V | 100.20% |
| 3 | L-Histidine | Sigma-Aldrich, USA | PHR1108 | Pharmaceutical Secondary Standard, certified reference material | 99.90% |
| 4 | L-Phenylalanine | Sigma-Aldrich, USA | PHR1100 | Pharmaceutical Secondary Standard, certified reference material | 99.90% |
| 5 | D-(+)-Glucose | Sigma-Aldrich, USA | PHR1000 | Pharmaceutical Secondary Standard, certified reference material | 99.50% |
| 6 | L-Aspartic acid | Sigma-Aldrich, USA | PHR1104 | Pharmaceutical Secondary Standard, certified reference material | 99.60% |
| 7 | L-Valine | Sigma-Aldrich, USA | PHR1172 | Pharmaceutical Secondary Standard, certified reference material | 98.90% |
| 8 | L-Alanine | Sigma-Aldrich, USA | PHR1110 | Pharmaceutical Secondary Standard, certified reference material | 98.90% |
| 9 | L-Serine | Sigma-Aldrich, USA | PHR1103 | Pharmaceutical Secondary Standard, certified reference material | 99.90% |
| 10 | L-carnitine | EDQM Council of Europe, France | L0399900 | European pharmacopoeia (ep) reference standard | NA |
| 11 | L-Glutamine | Sigma-Aldrich, USA | PHR1125 | Pharmaceutical Secondary Standard, certified reference material | 100.10% |
| 12 | Creatinine | Sigma-Aldrich, USA | PHR1462 | Pharmaceutical Secondary Standard, certified reference material | 99.80% |
| 13 | L-Proline | Sigma-Aldrich, USA | PHR1332 | Pharmaceutical Secondary Standard, certified reference material | 99.99% |
| 14 | Betaine | Sigma-Aldrich, SWITZERLAND | 30056 | Pharmaceutical Secondary Standard, certified reference material | 99.30% |
| 15 | L-Glycine | Sigma-Aldrich, SWITZERLAND | 76524 | Certified reference material | 99.80% |
| 16 | L-Arginine | Sigma-Aldrich, USA | PHR1106 | Pharmaceutical Secondary Standard, certified reference material | 99.60% |
| 17 | L-Threonine | Sigma-Aldrich, USA | PHR1242 | Pharmaceutical Secondary Standard, certified reference material | 99.96% |
| 18 | L-tryptophan | Sigma-Aldrich, USA | PHR1176 | Pharmaceutical Secondary Standard, certified reference material | 99.96% |
| 19 | L-Lysine | Sigma-Aldrich, SWITZERLAND | 23128 | Analytical standard | 100.00% |
| 20 | L-Glutamic acid | Sigma-Aldrich, USA | PHR1107 | Pharmaceutical Secondary Standard, certified reference material | 99.70% |
| 21 | L-Asparagine | Sigma-Aldrich, SWITZERLAND | 51363 | Certified reference material | 99.60% |
| 22 | Hypoxanthine | Sigma-Aldrich, China | H9377 | Analytical standard | 100.00% |

**Supplemental Table S2. Reported clinical significance of metabolites used to prepare two artificial groups of plasma samples.**

| **Metabolite** | **Clinical Significance** | **Supplemental References** |
| --- | --- | --- |
| L-Leucine | Diabetes, Huntington disease, pancreatic cancer | [47–49] |
| Creatine | Mitochondrial diseases, congenital urea cycle defects, kidney and liver diseases, cancer processes, thyroid defects, and autism, cardiac injury, guanidinoacetate methyltransferase deficiency | [32, 50, 51] |
| L-Histidine | Inflammatory Bowel Disease, renal cell carcinoma, knee osteoarthritis | [52–54] |
| L-Phenylalanine | Diabetes, Parkinson's disease, liver disease, gastroesophageal malignancy | [55–58] |
| D-(+)-Glucose | Knee osteoarthritis, diabetes mellitus, obesity, pancreatitis, | [54, 59–61] |
| L-Aspartic Acid | Cardiovascular Diseases, rheumatic diseases, Colorectal Cancer | [62–64] |
| L-Valine | Pancreatic cancer, diabetes, maple syrup urine disease | [47, 55, 64–66] |
| L-Alanine | Nonalcoholic fatty liver disease, mitochondrial diseases, Alzheimer’s disease | [67–71] |
| L-Serine | Huntington’s disease, Alzheimer's disease, schizophrenia, acute ischemic stroke progression | [72–76] |
| L-Carnitine | Knee osteoarthritis, Alzheimer's disease, autism spectrum disorder, acute myocardial infarction | [54, 75, 77–79] |
| L-Glutamine | Huntington’s disease, autism spectrum disorder, short bowel syndrome, predicting diabetic retinopathy, pediatric chronic intestinal pseudo-obstruction, sickle cell disease | [72, 80–84] |
| Creatinine | amyotrophic lateral sclerosis, kidney disease | [85, 86] |
| L-Proline | Breast cancer, pancreatic cancer, acute respiratory distress syndrome, type 2 diabetic coronary heart diseases | [87–90] |
| Betaine | Acute ischemic stroke progression, cardiovascular diseases, acute ischemic stroke progression, renal cell carcinoma | [76, 90–92] |
| L-Glycine | Chronic obstructive pulmonary disease, autism, Parkinson's disease | [80, 91–94] |
| L-Arginine | Breast cancer, hepatocellular carcinoma, chronic kidney disease, Jaundice syndrome, Alzheimer’s Disease | [87, 95–98] |
| L-Threonine | Huntington’s disease, acute respiratory distress syndrome, hepatocellular carcinoma, prostate cancer | [72, 99, 100] |
| L-Tryptophan | Inflammatory bowel disease, renal cell carcinoma, gastroesophageal malignancy, Alzheimer’s disease, Lyme disease, major depressive disorder | [52, 58, 98, 101, 102] |
| L-Lysine | Huntington’s disease, inborn errors, coronary artery disease, diabetic retinopathy | [72, 103–105] |
| L-Glutamic Acid | Pediatric chronic intestinal pseudo-obstruction, sickle cell disease, disorders of the nervous system | [84, 106, 107] |
| L-Asparagine | Huntington’s disease, ovarian cancer, Psoriasis | [72, 108, 109] |
| Hypoxanthine | Energy metabolism in intestinal epithelial cells, cardiac ischemia, Tuberculosis, non-Hodgkin lymphoma, myocardial infarction | [110–114] |

**Supplemental Table S3. The *snthresh* value used in recent 20 metabolomics studies.**

| **No.** | **Sample** | **Year** | ***snthresh* value** | **Supplemental Reference** |
| --- | --- | --- | --- | --- |
| 1 | serum | 2018 | 3 | [12] |
| 2 | plasma | 2017 | 5 | [8] |
| 3 | plasma | 2015 | 5 | [115] |
| 4 | plasma | 2015 | 5 | [116] |
| 5 | serum | 2016 | 5 | [117] |
| 6 | serum | 2016 | 5 | [118] |
| 7 | serum | 2013 | 5 | [119] |
| 8 | serum | 2017 | 5 | [120] |
| 9 | plasma | 2016 | 5 | [121] |
| 10 | plasma | 2015 | 5 | [122] |
| 11 | plasma | 2018 | 5 | [123] |
| 12 | plasma | 2016 | 6 | [124] |
| 13 | plasma | 2013 | 6 | [125] |
| 14 | serum | 2019 | 6 | [126] |
| 15 | plasma | 2018 | 6 | [127] |
| 16 | plasma | 2016 | 10 | [128] |
| 17 | plasma | 2017 | 10 | [129] |
| 18 | serum | 2017 | 10 | [130] |
| 19 | serum | 2016 | 10 | [131] |
| 20 | serum | 2018 | 20 | [132] |

**Supplemental Table S4. Summary of 165 false positive biomarkers among the 187 putative biomarkers discovered by comparing the metabolomic profiles of plasma samples mimicking those collected from diseased subjects (n = 12) and non-diseased subjects (n = 12). The metabolomic features were extracted using a *snthresh* value = 5. The annotation level of a metabolomic feature is described according to the Metabolomics Standards Initiative (MSI).**

| **Identity** | **Metabolite standards,**  **Set A or Set B** | **Retention time** | **Observed m/z** | **Normalized signal intensity (mean ± SD)** | | | **Fold-change  (Diseased /Non-diseased)** | **CAMERA annotation** | **Annotation level** |
| --- | --- | --- | --- | --- | --- | --- | --- | --- | --- |
|  |  |  |  | **Diseased** | **Non-diseased** | **BH adjusted *P*-value** |  |  |  |
| in source loss of HCOOH+NH_3_ from Val | A | 7.3 | 55.05455 | 1.2E+06±2.7E+05 | 3.7E+05±5.8E+04 | 7.8E-09 | 3.2 | [M+H-NH_3_]^+^ 71.0736 | LEVEL 1 |
| Allylamine | Neither | 6.3 | 58.06541 | 2.1E+06±1.2E+05 | 2.3E+06±2.0E+05 | 3.7E-02 | 0.9 | -- | LEVEL 3 |
| in source loss of CH_2_COOH from betaine | B | 7.7 | 59.07321 | 1.7E+06±2.8E+05 | 7.7E+06±5.5E+05 | 3.5E-18 | 0.2 | -- | LEVEL 1 |
| Urea | Neither | 2.2 | 61.03984 | 3.2E+08±3.7E+07 | 3.8E+08±4.4E+07 | 2.0E-02 | 0.9 | -- | LEVEL 1 |
| in-source loss of CH_5_N_3_+HCOOH from Arg | B | 10.5 | 70.06515 | 2.1E+06±3.0E+05 | 3.4E+06±2.2E+05 | 4.1E-10 | 0.6 | [M+H-HCOOH]^+^ 115.063 | LEVEL 1 |
| in-source loss of HCOOH from Pro | B | 7.8 | 70.06520 | 1.5E+07±1.4E+06 | 5.7E+07±1.1E+07 | 6.9E-11 | 0.3 | [M+H]+ 69.0583 [M+H-HCOOH]^+^ 115.064 | LEVEL 1 |
| In source loss of HCOOH from Thr | B | 8.9 | 74.06004 | 1.1E+06±9.6E+05 | 5.0E+06±1.2E+06 | 1.7E-07 | 0.2 | -- | LEVEL 1 |
| in source loss of HCOOH+NH_3_ from Gln | A | 9.4 | 84.04436 | 7.8E+06±2.0E+06 | 5.3E+06±1.6E+06 | 1.1E-02 | 1.5 | [M+H-HCOOH]^+^ 129.043 | LEVEL 1 |
| in source loss of HCOOH from Asn | B | 9.5 | 87.05528 | 2.1E+05±5.4E+04 | 9.6E+05±1.8E+05 | 5.0E-11 | 0.2 | [M+H]+ 86.048 | LEVEL 1 |
| in source loss of HCOOH Asp | A | 9.4 | 88.03927 | 1.3E+06±2.5E+05 | 6.4E+05±2.4E+05 | 1.4E-05 | 2.0 | [M+H-COCH_2_]^+^ 129.043 | LEVEL 1 |
| in source loss of C_2_H_3_NO_2_ from Phe | A | 5.4 | 93.06988 | 8.0E+05±8.6E+04 | 2.2E+05±3.1E+04 | 1.1E-14 | 3.6 | [3M+H+Na]2^+^ 54.0486 | LEVEL 1 |
| in source loss of HCOOH from Glu | B | 8.9 | 102.05494 | 1.8E+06±3.1E+05 | 5.7E+06±1.1E+06 | 3.5E-10 | 0.3 | -- | LEVEL 1 |
| in source loss of HCOOH+NH_3_ from Phe | A | 5.4 | 103.05427 | 2.7E+06±4.6E+05 | 8.9E+05±2.0E+05 | 2.4E-10 | 3.0 | [M+H]^+^ 102.047 | LEVEL 1 |
| [M+2Na-H-C_4_H_8_O_4_]^+^ of Glucose | A | 7.6 | 104.99231 | 5.8E+06±5.7E+05 | 2.3E+06±3.5E+05 | 3.2E-13 | 2.5 |  | LEVEL 1 |
| in source loss of HCOOH from His | A | 10.4 | 110.07123 | 5.0E+06±8.5E+05 | 1.7E+06±2.0E+05 | 1.0E-10 | 3.0 | -- | LEVEL 1 |
| in source loss of CO+N_2_H_4_ from Arg | B | 10.5 | 115.08659 | 2.6E+06±2.0E+05 | 3.4E+06±5.7E+05 | 3.2E-04 | 0.7 | -- | LEVEL 1 |
| in source loss of CH_5_N_3_ from Arg | B | 10.5 | 116.07057 | 9.2E+06±7.7E+05 | 1.3E+07±9.8E+05 | 4.6E-10 | 0.7 | [M+H]^+^ 115.063 [M+H-NH_3_]^+^ 132.09 | LEVEL 1 |
| [M+2Na-H-C_3_H_8_O_4_]+ of Glucose | A | 7.6 | 116.99231 | 8.6E+05±9.4E+04 | 2.5E+05±8.0E+04 | 9.2E-13 | 3.4 | -- | LEVEL 1 |
| in source loss of H_2_O from Glu | B | 9.0 | 129.06582 | 9.8E+06±7.5E+05 | 1.1E+07±1.1E+06 | 2.4E-03 | 0.9 | [M+H]^+^ 128.059 | LEVEL 1 |
| in source loss of NH_3_ and H_2_O from Phe | A | 5.5 | 131.04917 | 2.3E+06±2.4E+05 | 7.3E+05±1.4E+05 | 4.1E-14 | 3.2 | -- | LEVEL 1 |
| ornithine | Neither | 10.5 | 133.09708 | 1.1E+07±7.7E+05 | 1.6E+07±4.7E+06 | 8.7E-03 | 0.7 | [M+H+NH_3_]^+^ 115.063  [M+H]^+^ 132.09 | LEVEL 1 |
| M+2 of Leu | A | 5.9 | 134.10610 | 6.9E+05±7.6E+04 | 1.9E+05±2.9E+04 | 1.7E-14 | 3.6 | -- | LEVEL 1 |
| in source loss of C_3_H_6_O_3_ from Glucose [M+Na]^+^ | A | 7.6 | 135.00288 | 3.1E+06±2.9E+05 | 1.3E+06±2.8E+05 | 7.3E-12 | 2.4 | [M+2Na]^2+^ 224.027 | LEVEL 1 |
| [M+Na-H_2_O]^+^ of Creatine | A | 8.7 | 136.04812 | 2.9E+07±5.5E+06 | 1.3E+07±4.2E+06 | 7.4E-07 | 2.2 | -- | LEVEL 1 |
| M+1 ^13^C of Hypoxanthine | B | 2.7 | 138.04907 | 4.9E+06±6.4E+05 | 1.1E+07±1.9E+06 | 1.0E-08 | 0.5 | -- | LEVEL 1 |
| in source loss of CH_4_NO_2_ from Trp | B | 5.3 | 143.07271 | 1.2E+05±6.0E+04 | 6.0E+05±2.4E+05 | 4.8E-06 | 0.2 | -- | LEVEL 1 |
| in source loss of C_2_H_5_NO Trp | B | 5.3 | 146.06003 | 2.1E+06±1.2E+06 | 8.5E+06±2.7E+06 | 9.7E-07 | 0.2 | -- | LEVEL 1 |
| In source loss of H_2_O from [M+Na]^+^ Gln | A | 9.4 | 151.04772 | 3.1E+07±3.6E+06 | 1.2E+07±2.2E+06 | 3.3E-12 | 2.7 | -- | LEVEL 1 |
| In source loss of NH_3_ from[ M+Na]^+^ Gln | A | 9.4 | 152.03171 | 1.9E+07±1.9E+06 | 7.2E+06±1.4E+06 | 4.7E-13 | 2.7 | [M+Na]^+^ 129.043 | LEVEL 1 |
| ^13^C M+1 of [M+K]^+^ of betaine | B | 7.7 | 157.04563 | 3.7E+05±1.8E+05 | 1.1E+06±4.7E+05 | 4.0E-04 | 0.3 | -- | LEVEL 1 |
| ^41^K M+2 of [M+K]^+^ of betaine | B | 7.7 | 158.04032 | 4.7E+05±2.6E+05 | 1.4E+06±5.8E+05 | 1.6E-04 | 0.3 | -- | LEVEL 1 |
| in source loss of HCOOH from Trp | B | 5.3 | 159.09165 | 1.8E+06±9.0E+04 | 5.1E+06±8.9E+05 | 1.7E-10 | 0.4 | [M+H]^+^ 158.084 | LEVEL 1 |
| In source loss of C_2_H_4_O_2_ from [M+2Na-H]^+^ Glucose | A | 7.6 | 165.01344 | 3.4E+06±2.7E+05 | 1.3E+06±1.8E+05 | 4.1E-15 | 2.7 | -- | LEVEL 1 |
| [M+Na]^+^ of Gln | A | 9.4 | 169.05825 | 1.5E+08±2.0E+07 | 5.7E+07±1.1E+07 | 4.2E-11 | 2.6 | [M+Na+NH_3_]^+^ 129.043 | LEVEL 1 |
| [M+Na]^+^ of Lys | B | 10.5 | 169.09473 | 3.7E+06±8.4E+05 | 1.3E+07±1.4E+06 | 2.4E-14 | 0.3 | [M+H]^+^ 168.087 | LEVEL 1 |
| M+1 ^13^C of Lys [M+Na]^+^ | B | 10.5 | 170.09239 | 1.1E+07±7.5E+05 | 1.3E+07±1.3E+06 | 1.2E-03 | 0.9 | -- | LEVEL 1 |
| M+2 ^41^K [M+K]^+^ Creatine | A | 8.7 | 172.03066 | 9.2E+05±2.6E+05 | 4.8E+05±2.4E+05 | 1.3E-03 | 1.9 | -- | LEVEL 1 |
| [M+K+Na-H]^+^ Val | A | 7.3 | 178.02406 | 4.1E+06±1.7E+05 | 2.3E+06±4.0E+05 | 2.1E-11 | 1.8 | [M+K+NaCOOH]^+^ 71.0736 | LEVEL 1 |
| [M+Na]^+^ of His | A | 10.4 | 178.05847 | 2.7E+07±2.9E+06 | 7.6E+06±1.2E+06 | 1.5E-14 | 3.5 | [M+H]^+^ 177.051 | LEVEL 1 |
| Arg [M+Na-H_2_O]^+^ | B | 10.4 | 179.09006 | 1.1E+06±1.6E+05 | 2.7E+06±4.3E+05 | 4.2E-10 | 0.4 | -- | LEVEL 1 |
| Arg [M+Na-NH_3_]^+^ | B | 10.4 | 180.07399 | 3.2E+05±5.0E+04 | 8.5E+05±1.7E+05 | 5.1E-09 | 0.4 | -- | LEVEL 1 |
| [M+K ]^+^ of Gln | A | 9.4 | 185.03223 | 1.1E+07±8.1E+05 | 5.9E+06±8.5E+05 | 3.8E-11 | 1.8 | [M+K+NH_3]_^+^ 129.043 | LEVEL 1 |
| Ecgonine | Neither | 6.9 | 186.11242 | 5.6E+06±5.2E+05 | 6.2E+06±4.6E+05 | 3.7E-02 | 0.9 | -- | LEVEL 2 |
| C_16_H_16_O_11_ | Neither | 1.2 | 212.01975 | 3.7E+05±4.9E+04 | 4.4E+05±5.4E+04 | 1.9E-02 | 0.8 | -- | LEVEL 3 |
| N-*a*-Acetyl-L-arginine | Neither | 8.6 | 217.12944 | 5.0E+05±8.4E+04 | 3.6E+05±1.0E+05 | 8.3E-03 | 1.4 | -- | LEVEL 3 |
| [M+K]^+^ of Glucose | A | 7.3 | 219.02640 | 2.5E+07±7.7E+06 | 1.1E+07±2.6E+06 | 4.3E-05 | 2.3 | -- | LEVEL 1 |
| [M+Na]^+^ of Trp | B | 5.4 | 227.07895 | 5.3E+06±1.4E+06 | 1.1E+07±1.4E+06 | 7.2E-09 | 0.5 | -- | LEVEL 1 |
| [M+2Na-H+HCOONa]^+^ of Val | A | 7.4 | 230.03719 | 6.8E+05±1.7E+05 | 2.7E+05±6.2E+04 | 1.0E-06 | 2.5 | [M+H-HCOOH]^+^ 275.036 | LEVEL 1 |
| Ergothioneine | Neither | 8.4 | 230.09562 | 5.2E+05±7.9E+04 | 6.2E+05±6.5E+04 | 7.7E-03 | 0.8 | -- | LEVEL 2 |
| [M+2Na-H+NaCl]^+^ of Leu | A | 6.0 | 234.02424 | 1.4E+06±3.0E+05 | 8.5E+05±1.7E+05 | 2.8E-04 | 1.6 | -- | LEVEL 1 |
| [2M+H]^+^ Betaine | B | 7.7 | 235.16510 | 1.1E+04±1.1E+04 | 2.1E+06±3.2E+05 | 5.9E-15 | 0.0 | [M+H]^+^ 234.158 | LEVEL 1 |
| taurine [M+2Na-H+HCOONa]^+^ | Neither | 7.4 | 237.97314 | 4.1E+05±1.3E+05 | 7.0E+05±9.9E+04 | 2.2E-05 | 0.6 | [M+H+NH_3]_^+^ 219.937 | LEVEL 2 |
| Trp [M+K]^+^ | B | 5.4 | 243.05294 | 1.2E+06±4.8E+05 | 1.9E+06±4.1E+05 | 2.9E-03 | 0.6 | [2M+K]^+^ 102.047 | LEVEL 1 |
| C_16_H_18_O_2_ | Neither | 2.1 | 243.13384 | 4.2E+05±4.0E+04 | 4.7E+05±5.0E+04 | 3.5E-02 | 0.9 | -- | LEVEL 3 |
| [M+2Na-H+HCOONa]^+^ of Creatine | A | 8.7 | 244.02788 | 7.4E+05±2.1E+05 | 4.8E+05±1.7E+05 | 1.3E-02 | 1.5 | -- | LEVEL 1 |
| Glucose [M+Na+ACN]^+^ | A | 5.9 | 244.07912 | 3.8E+06±4.9E+05 | 1.6E+06±2.3E+05 | 3.2E-11 | 2.4 | [M+Na]^+^ 221.09 | LEVEL 1 |
| [2M+3Na-2H]+ of Ala | A | 8.6 | 245.04842 | 2.5E+06±7.1E+05 | 1.2E+06±3.8E+05 | 8.6E-05 | 2.1 | [2M+Na]^+^ 111.03 [M+Na]^+^ 222.059 | LEVEL 1 |
| Gln [M+2Na-H+HCOONa]^+^ | A | 9.3 | 259.02758 | 5.5E+05±1.0E+05 | 7.3E+05±1.8E+05 | 3.4E-02 | 0.8 | [M+2Na]^2+^ 472.075 | LEVEL 1 |
| [M+2Na-H+NaCl]^+^ of Phe | A | 5.5 | 268.00854 | 1.8E+06±7.4E+05 | 6.4E+05±2.8E+05 | 3.1E-04 | 2.8 | -- | LEVEL 1 |
| [M+Na+HCOONa]^+^ Glucose | A | 7.6 | 271.03999 | 1.9E+06±2.4E+05 | 5.6E+05±1.6E+05 | 3.3E-12 | 3.4 | [M+H+HCOOH]^+^ 224.027 | LEVEL 1 |
| [2Gly(spiked)+Thr(spiked)+4Na-3H+]^+^ | B | 9.1 | 358.05703 | 1.0E+05±1.9E+04 | 1.0E+06±1.6E+05 | 4.1E-14 | 0.1 | [M+K]^+^ 319.094 [M+Na]^+^ 335.068 [M+H]^+^ 357.05 | LEVEL 1 |
| [Lys(spiked)+Arg(spiked)+2Na-H]^+^ | B | 10.5 | 365.18797 | 1.2E+05±4.2E+04 | 8.0E+05±1.6E+05 | 3.6E-11 | 0.2 | -- | LEVEL 1 |
| 1-Methyladenosine | Neither | 7.3 | 282.11960 | 1.5E+06±1.3E+05 | 1.7E+06±1.2E+05 | 1.7E-02 | 0.9 | -- | LEVEL 2 |
| [Pro(endogenous)+Betaine(spiked)+2Na-H]^+^ | B | 7.8 | 277.11343 | 2.4E+05±1.9E+05 | 1.6E+06±4.3E+05 | 1.5E-08 | 0.2 | -- | LEVEL 1 |
| [2M+3Na-2H+HCOONa]^+^ of Gly | B | 9.1 | 285.00443 | 2.7E+06±7.9E+05 | 6.2E+06±1.4E+06 | 1.2E-06 | 0.4 | [M+K]^+^ 246.041 | LEVEL 1 |
| Arg [M+2Na-H+HCOONa]^+^ | B | 10.4 | 287.06989 | 1.8E+05±7.2E+04 | 9.7E+05±2.8E+05 | 2.8E-08 | 0.2 | [M+K]^+^ 248.107 [M+H]^+^ 286.062 | LEVEL 1 |
| in source loss of CH2N2 from creatine [2M+3Na-2H]^+^ | A | 8.7 | 287.06996 | 2.4E+06±2.4E+05 | 8.8E+05±1.1E+05 | 6.6E-14 | 2.7 | [M+K]^+^ 248.107 | LEVEL 1 |
| [Thr+2Na-H+NaCl+HCOONa]^+^ | B | 8.9 | 289.97524 | 3.9E+05±1.1E+05 | 6.0E+05±2.1E+05 | 2.7E-02 | 0.7 | -- | LEVEL 1 |
| [M+2Na-H+2NaCl]^+^ of Leu | A | 5.9 | 291.98288 | 7.2E+05±1.5E+05 | 3.3E+05±9.8E+04 | 1.2E-06 | 2.2 | -- | LEVEL 1 |
| [Glu(spiked)+Asn(spiked)+3Na-2H+]^+^ | B | 9.5 | 346.05940 | 3.1E+05±1.1E+05 | 1.4E+06±4.0E+05 | 2.9E-08 | 0.2 | [M+H]^+^ 345.052 | LEVEL 1 |
| M+2 ^37^Cl of [M+2Na-H+2NaCl]^+^ of Leu | A | 5.9 | 293.97993 | 4.3E+05±9.2E+04 | 1.9E+05±5.5E+04 | 1.2E-06 | 2.2 | -- | LEVEL 1 |
| [M+2Na-H+2HCOONa]^+^ of Pro | B | 7.8 | 296.00923 | 4.8E+06±1.4E+06 | 1.6E+07±4.3E+06 | 2.8E-07 | 0.3 | [M+H-HCOOH]^+^ 341.007 | LEVEL 1 |
| [Asp+Na_2_HPO_4_+Na]^+^ | A | 10.0 | 297.96738 | 7.2E+05±2.8E+05 | 3.1E+05±1.5E+05 | 8.7E-04 | 2.3 | [M+H]^+^ 296.96 | LEVEL 1 |
| Val [M+2Na-H+2HCOONa]^+^ | A | 7.4 | 298.02482 | 5.6E+06±1.6E+06 | 2.4E+06±3.9E+05 | 3.9E-06 | 2.4 | [M+Na]^+^ 275.036 [M+H-HCOOH]^+^ 343.023 | LEVEL 1 |
| [Thr+2Na-H+2HCOONa]^+^ | B | 8.9 | 300.00407 | 3.6E+06±1.2E+06 | 6.4E+06±2.1E+06 | 2.4E-03 | 0.6 | [M+2Na]^2+^ 554.03 | LEVEL 1 |
| [M+2Na-H+NaCl]^+^ of Trp | B | 5.3 | 307.01944 | 1.2E+06±3.4E+05 | 2.7E+06±1.0E+06 | 3.2E-04 | 0.4 | -- | LEVEL 1 |
| [2M+3Na-2H+HCOONa]^+^ of Ala | A | 8.6 | 313.03572 | 7.2E+06±1.8E+06 | 4.2E+06±1.1E+06 | 3.4E-04 | 1.7 | [M+Na+NaCOOH]^+^ 222.059 | LEVEL 1 |
| [Lys(spiked)+ornithine(endogenous)+4Na-3H]^+^ | B | 10.5 | 513.23557 | 2.9E+05±6.2E+04 | 1.1E+06±2.0E+05 | 1.3E-10 | 0.3 | -- | LEVEL 1 |
| [2Gln(endogenous)+Gly(spiked)+4Na-3H]^+^ | B | 9.3 | 456.10470 | 1.4E+05±6.5E+04 | 4.7E+05±1.3E+05 | 5.7E-07 | 0.3 | -- | LEVEL 1 |
| [M+2Na-H+2NaCl]^+^ of Phe | A | 5.5 | 325.96720 | 7.1E+05±3.6E+05 | 2.4E+05±1.6E+05 | 2.1E-03 | 3.0 | -- | LEVEL 1 |
| [M+2Na-H+2HCOONa]^+^ of Gln | A | 9.3 | 327.01501 | 9.8E+05±1.8E+05 | 1.6E+06±4.5E+05 | 1.9E-03 | 0.6 | [M+H-C_6_H_10_O_4_]^+^ 472.075 | LEVEL 1 |
| [M+3Na-2H+HCOONa]^+^ of Lys | B | 10.5 | 327.05121 | 1.2E+06±3.6E+05 | 2.2E+06±7.5E+05 | 1.9E-03 | 0.5 | -- | LEVEL 1 |
| [2M+3Na-2H]^+^ of creatine | A | 8.7 | 329.09149 | 1.8E+06±1.8E+05 | 6.5E+05±9.5E+04 | 5.7E-14 | 2.8 | -- | LEVEL 1 |
| [2M+3Na-2H]^+^ of creatine | A | 8.7 | 329.09159 | 2.1E+06±2.0E+05 | 4.5E+05±1.3E+05 | 3.3E-15 | 4.6 | [M+H-HCOOH]^+^ 374.09 | LEVEL 1 |
| [Val+2K-H+2HCOONa]^+^ | A | 7.3 | 329.97263 | 6.1E+05±1.0E+05 | 4.5E+05±1.1E+05 | 8.4E-03 | 1.3 | [M+Na]^+^ 306.983 | LEVEL 1 |
| [M+2Na-H+2HCOONa]^+^ of His | A | 10.4 | 336.01519 | 1.4E+06±5.2E+05 | 4.8E+05±1.9E+05 | 5.0E-05 | 2.9 | [M+H-HCOOH]^+^ 381.013 | LEVEL 1 |
| [2M+2Na-H]^+^ of Gln | A | 9.4 | 337.10917 | 2.8E+06±6.1E+05 | 6.9E+05±2.0E+05 | 1.0E-09 | 4.1 | [M+H]^+^ 336.102 | LEVEL 1 |
| Taurine [M+2K-H+2HCOONa]^+^ | Neither | 7.3 | 337.90806 | 3.2E+05±6.8E+04 | 5.9E+05±2.0E+05 | 8.1E-04 | 0.5 | [M+Na+NaCOOH]^+^ 246.932 | LEVEL 2 |
| [Betaine+C_7_H_13_NO_2_+Na]^+^ | B | 7.7 | 283.16277 | 4.9E+05±2.4E+05 | 1.7E+06±7.1E+05 | 1.1E-04 | 0.3 | [M+H]^+^ 282.156 | LEVEL 1 |
| [creatine(endogenous)+2Thr(spiked)+4Na-3H]^+^ | B | 8.8 | 458.12090 | 1.2E+05±2.7E+04 | 3.4E+05±7.5E+04 | 2.0E-08 | 0.4 | -- | LEVEL 1 |
| [Arg(spiked)+background C_5_H_11_NO_2_+H]^+^ | B | 10.4 | 292.19744 | 2.4E+05±9.6E+04 | 5.5E+05±1.8E+05 | 2.0E-04 | 0.4 | -- | LEVEL 1 |
| [Lys(spiked)+ornithine(endogenous)+3Na-2H]^+^ | B | 10.5 | 345.14813 | 7.2E+05±1.5E+05 | 1.5E+06±3.0E+05 | 1.0E-06 | 0.5 | [M+H-HCOOH]^+^ 390.146 | LEVEL 1 |
| [M+2Na-H+3NaCl]^+^ of Leu | A | 5.9 | 349.94152 | 8.0E+05±2.1E+05 | 4.7E+05±1.6E+05 | 1.8E-03 | 1.7 | -- | LEVEL 1 |
| M+2 of [M+2Na-H+3NaCl]^+^ of Leu | A | 5.9 | 351.93846 | 7.7E+05±1.8E+05 | 4.0E+05±1.4E+05 | 8.3E-05 | 1.9 | -- | LEVEL 1 |
| [Ala(spiked)+creatine(spiked)+3Na-2H+HCOONa]^+^ | A | 8.7 | 355.05682 | 1.9E+06±3.9E+05 | 8.4E+05±1.6E+05 | 6.8E-08 | 2.3 | [M+K+NaCOOH]^+^ 248.107 | LEVEL 1 |
| [M+2Na-H+2HCOONa]^+^ of Arg | B | 10.4 | 355.05742 | 3.1E+05±1.2E+05 | 1.5E+06±4.4E+05 | 7.1E-08 | 0.2 | [M+K+CF_3_COOH]^+^ 182.102 [M+K+NaCOOH]^+^ 248.107 [M+Na+HCOOH]+ 286.062 | LEVEL 1 |
| [3M+4Na-3H]^+^ of Ala | A | 8.6 | 356.07772 | 3.3E+06±5.4E+05 | 1.0E+06±1.6E+05 | 3.2E-11 | 3.2 | [3M+Na]+ 111.03 | LEVEL 1 |
| [Asn(spiked) +Gln(spiked)+3Na-2H]^+^ | B | 9.5 | 345.07545 | 6.7E+05±1.2E+05 | 9.7E+05±1.8E+05 | 3.1E-04 | 0.7 | [M+H-C_6_H_10_O_4_]^+^ 490.133 [M+H-C_6_H_8_O_6_]^+^ 520.097 | LEVEL 1 |
| ^18^O [2M+3Na-2H]^+^ of Gln | A | 9.4 | 361.05912 | 1.3E+06±3.9E+05 | 3.0E+06±1.0E+06 | 1.1E-04 | 0.4 | -- | LEVEL 1 |
| [2M+3Na-2H+NaCl]^+^ of Thr | B | 8.9 | 363.02784 | 9.6E+04±4.1E+04 | 5.2E+05±1.3E+05 | 1.8E-09 | 0.2 | [M+K]^+^ 324.065 [M+Na]^+^ 340.039 | LEVEL 1 |
| [M+2Na-H+3HCOONa]^+^ of Pro | B | 7.8 | 363.99658 | 4.3E+05±1.5E+05 | 1.4E+06±3.6E+05 | 8.4E-08 | 0.3 | [M+Na]^+^ 341.007 [M+H-HCOOH]^+^ 408.995 | LEVEL 1 |
| [Ala(spiked]+Thr(endogenous)+3Na-2H]^+^ | A | 8.8 | 275.05891 | 4.2E+05±6.4E+04 | 3.4E+05±5.7E+04 | 2.5E-02 | 1.2 | [M+H-HCOOH]^+^ 320.057 | LEVEL 1 |
| [M+2Na-H+3HCOONa]^+^ of Val | A | 7.4 | 366.01233 | 5.8E+05±1.9E+05 | 2.2E+05±8.6E+04 | 3.5E-05 | 2.6 | [M+Na+NaCOOH]^+^ 275.036  [M+Na]^+^ 343.023 | LEVEL 1 |
| [Ala(spiked)+Thr(endogenous)+3Na-2H+HCOONa]^+^ | A | 8.8 | 343.04613 | 7.7E+05±1.1E+05 | 5.4E+05±6.0E+04 | 8.7E-06 | 1.4 | [M+Na]^+^ 320.057 | LEVEL 1 |
| [2M+K+Na-H]^+^ of Gln | A | 9.3 | 375.06494 | 6.4E+05±5.4E+04 | 2.7E+05±5.0E+04 | 6.0E-13 | 2.4 | [M+K]^+^ 336.103 | LEVEL 1 |
| C_21_H_38_O_10_ | Neither | 1.2 | 379.21345 | 3.3E+05±4.7E+04 | 4.0E+05±6.6E+04 | 3.9E-02 | 0.8 | -- | LEVEL 3 |
| [2M+3Na-2H+2HCOONa]^+^ of Ala | A | 8.6 | 381.02295 | 9.2E+05±3.1E+05 | 5.8E+05±2.4E+05 | 2.9E-02 | 1.6 | -- | LEVEL 1 |
| [3Gly+4Na-3H+HCOONa]^+^ | B | 9.1 | 382.01810 | 1.4E+05±5.4E+04 | 5.3E+05±1.5E+05 | 1.4E-07 | 0.3 | [M+K+NaCOOH]^+^ 275.068 | LEVEL 1 |
| [2Val+2Na+K-2H+HCOONa]^+^ | B | 7.3 | 385.07184 | 6.9E+05±1.0E+05 | 2.1E+05±4.6E+04 | 1.4E-11 | 3.3 | [M+K]^+^ 346.109 | LEVEL 1 |
| [Taurine(endogenous)+Val(spiked)+2Na+K-2H]^+^ | A | 7.3 | 325.02035 | 6.9E+05±1.7E+05 | 4.7E+05±1.9E+05 | 3.3E-02 | 1.5 | [M+Na]^+^ 302.031 | LEVEL 1 |
| [2M+3Na-2H]^+^ of Phe | A | 5.5 | 397.11080 | 1.4E+06±5.1E+05 | 9.0E+04±7.9E+04 | 9.8E-08 | 15.7 | [M+H]^+^ 396.104 | LEVEL 1 |
| [Lys(endogenous)+His(spiked)+3Na-2H]^+^ | A | 10.5 | 368.12769 | 9.1E+05±1.8E+05 | 6.1E+05±1.1E+05 | 3.1E-04 | 1.5 | -- | LEVEL 1 |
| [Betaine+226.95+HCOONa]^+^ | B | 7.7 | 412.01786 | 5.5E+04±2.5E+04 | 5.0E+05±1.6E+05 | 2.0E-08 | 0.1 | -- | LEVEL 1 |
| [2M+3Na-2H]^+^ of Arg | B | 10.4 | 415.17610 | 5.6E+04±1.9E+04 | 1.0E+06±3.0E+05 | 2.5E-09 | 0.1 | [M+H]^+^ 414.168 | LEVEL 1 |
| [Trp(endogenous)+Phe(spiked)+3Na-2H]^+^ | A | 5.4 | 436.12150 | 9.0E+05±2.5E+05 | 4.9E+05±2.3E+05 | 2.2E-03 | 1.8 | [M+Na+NH_3_]^+^ 396.104 | LEVEL 1 |
| [3M+4Na-3H+HCOONa]^+^ of Ala | A | 8.6 | 424.06518 | 1.1E+06±3.2E+05 | 3.5E+05±1.4E+05 | 2.4E-06 | 3.1 | [M+K]^+^ 385.102 | LEVEL 1 |
| [2M+3Na-2H+HCOONa]^+^ Lys | B | 10.5 | 427.15126 | 3.2E+05±1.3E+05 | 1.1E+06±3.0E+05 | 3.8E-07 | 0.3 | [M+H-CH_2_]^+^ 440.16 | LEVEL 1 |
| [M+2Na-H+4HCOONa]^+^ of Pro | B | 7.8 | 431.98409 | 9.6E+05±3.8E+05 | 2.8E+06±1.2E+06 | 2.0E-04 | 0.3 | [M+Na+NaCOOH]^+^ 341.007 [M+Na]^+^ 408.995 | LEVEL 1 |
| [M+2Na-H+4HCOONa]^+^ of Val | A | 7.4 | 433.99946 | 9.7E+05±2.6E+05 | 3.9E+05±1.3E+05 | 3.9E-06 | 2.5 | [M+Na+NaCOOH]^+^ 343.023 | LEVEL 1 |
| [Thr+2Na-H+4HCOONa]^+^ | B | 8.9 | 435.97875 | 4.5E+05±1.5E+05 | 7.0E+05±2.3E+05 | 2.3E-02 | 0.6 | [M+K]^+^ 397.016 [M+Na]^+^ 412.99 [M+H]^+^ 434.971 | LEVEL 1 |
| [Glu(endogenous)+Gln(spiked)+5Na-4H]^+^ | A | 9.4 | 550.10820 | 3.0E+06±7.9E+05 | 1.3E+06±4.1E+05 | 1.3E-05 | 2.2 | [M+K]^+^ 511.146 | LEVEL 1 |
| PE(P-16:0) | Neither | 2.4 | 438.29783 | 1.1E+06±1.1E+05 | 1.3E+06±2.2E+05 | 4.5E-02 | 0.8 | [M+Na+NaCOOH]^+^ 347.325 | LEVEL 3 |
| creatine adduct | A | 8.7 | 440.12159 | 9.7E+05±1.2E+05 | 1.5E+05±5.6E+04 | 1.0E-14 | 6.4 | -- | LEVEL 1 |
| [2Thr+3Na-2H+2HCOONa]^+^ | B | 8.9 | 441.04422 | 2.0E+05±7.7E+04 | 8.3E+05±2.7E+05 | 6.7E-07 | 0.2 | -- | LEVEL 1 |
| [5M+5Na-3H]2^+^ of Gln | A | 9.3 | 443.11606 | 1.6E+06±3.5E+05 | 2.9E+05±9.0E+04 | 1.8E-10 | 5.5 | [M+H-C_6_H_10_O_4_]^+^ 588.174 | LEVEL 1 |
| [3Gly+4Na-3H+2HCOONa]^+^ | B | 9.1 | 450.00561 | 1.7E+05±6.8E+04 | 5.3E+05±1.8E+05 | 9.4E-06 | 0.3 | [M+H-HCOOH]^+^ 495.004 | LEVEL 1 |
| [Citrulline(endogenous)+Gln(spiked)+3Na-2H]^+^ | A | 9.6 | 388.11758 | 3.9E+05±4.2E+04 | 1.6E+05±3.1E+04 | 1.1E-11 | 2.4 | [M+H]^+^ 387.11 | LEVEL 1 |
| [Ala(spiked)+creatine(spiked)+3Na-2H]^+^ | A | 8.7 | 423.04491 | 4.1E+05±1.2E+05 | 1.6E+05±6.5E+04 | 1.5E-05 | 2.5 | [M+K]^+^ 384.082 [M+Na]^+^ 400.056 [M+H]^+^ 422.038 | LEVEL 1 |
| Gly [M+2Na-H+5HCOONa]^+^ | A | 9.2 | 459.94005 | 6.1E+05±2.1E+05 | 3.7E+05±1.7E+05 | 2.3E-02 | 1.6 | [M+Na]^+^ 436.951 | LEVEL 1 |
| [3M+3Na-2H]^+^ of Creatine | A | 8.7 | 460.16115 | 4.1E+05±7.9E+04 | 4.2E+04±2.5E+04 | 5.9E-12 | 9.9 | -- | LEVEL 1 |
| double charge Gln adduct | A | 9.4 | 466.08296 | 5.2E+05±1.4E+05 | 2.1E+05±7.2E+04 | 5.8E-06 | 2.5 | -- | LEVEL 1 |
| [2M+3Na-2H]^+^ Trp | B | 5.3 | 475.13276 | 7.0E+05±1.8E+05 | 4.0E+06±1.0E+06 | 1.4E-09 | 0.2 | -- | LEVEL 1 |
| [2M+3Na-2H+HCOONa]^+^ of Arg | B | 10.4 | 483.16297 | 2.8E+04±2.3E+04 | 7.5E+05±2.6E+05 | 2.8E-08 | 0.0 | [M+Na+HCOOH]^+^ 414.168 | LEVEL 1 |
| [3M+4Na-3H+2HCOONa]^+^ of Ala | A | 8.6 | 492.05265 | 1.1E+06±2.8E+05 | 4.3E+05±1.2E+05 | 3.4E-06 | 2.5 | [M+K+NaCOOH]^+^ 385.102 | LEVEL 1 |
| [M+2Na-H+5HCOONa]^+^ of Pro | B | 7.8 | 499.97146 | 1.4E+06±5.2E+05 | 4.2E+06±1.3E+06 | 4.4E-06 | 0.3 | [M+Na+NaCOOH]^+^ 408.995 | LEVEL 1 |
| [M+2Na-H+5HCOONa]^+^ of Val | A | 7.4 | 501.98715 | 1.1E+06±2.3E+05 | 4.4E+05±8.7E+04 | 5.1E-08 | 2.5 | -- | LEVEL 1 |
| [2Thr+3Na-2H+3HCOONa]^+^ | B | 8.9 | 509.03162 | 1.5E+05±6.3E+04 | 5.7E+05±1.9E+05 | 2.3E-06 | 0.3 | [M+H-HCOOH]^+^ 554.03 | LEVEL 1 |
| [Ser(spiked)+Gln(spiked)+3Na-2H]^+^ | A | 9.4 | 318.06478 | 1.3E+06±4.7E+05 | 4.4E+05±8.3E+04 | 1.4E-05 | 3.0 | -- | LEVEL 1 |
| [Creatine(spiked)+2Ala(spiked)+4Na-3H]^+^ | A | 8.7 | 398.09939 | 1.6E+06±1.1E+05 | 3.9E+05±9.5E+04 | 1.1E-16 | 4.1 | -- | LEVEL 1 |
| [3Gly+4Na-3H+3HCOONa]^+^ | B | 9.1 | 517.99291 | 2.1E+05±9.0E+04 | 4.2E+05±1.5E+05 | 2.2E-03 | 0.5 | [M+Na]^+^ 495.004 | LEVEL 1 |
| [3M+4Na-3H]^+^ of Gln | A | 9.3 | 527.14204 | 1.7E+07±2.5E+06 | 5.5E+06±9.7E+05 | 1.2E-11 | 3.1 | -- | LEVEL 1 |
| [3M+4Na-3H]^+^ of Gln | A | 9.3 | 527.15103 | 2.4E+06±5.9E+05 | 6.7E+05±1.7E+05 | 1.8E-08 | 3.6 | [3M+2Na]^2+^ 336.103 | LEVEL 1 |
| [4M+5Na-4H+HCOONa]^+^ of Ala | A | 8.6 | 535.09491 | 6.5E+05±1.4E+05 | 1.0E+05±3.8E+04 | 7.4E-11 | 6.3 | [M+K]^+^ 496.132 [M+H]^+^ 534.088 | LEVEL 1 |
| [3M+3Na+K-3H]^+^ of Gln | A | 9.3 | 543.11589 | 6.3E+05±8.2E+04 | 2.3E+05±7.2E+04 | 2.5E-10 | 2.7 | [3M+2K]^2+^ 336.103 | LEVEL 1 |
| [Creatine(spiked)+3Ala(spiked)+5Na-4H]^+^ | A | 8.7 | 509.12927 | 3.0E+05±7.1E+04 | 3.1E+04±1.8E+04 | 1.5E-10 | 9.9 | -- | LEVEL 1 |
| [3M+4Na-3H+3HCOONa]^+^ of Ala | A | 8.6 | 560.03994 | 7.2E+05±2.1E+05 | 4.1E+05±1.2E+05 | 8.9E-04 | 1.8 | -- | LEVEL 1 |
| [M+2Na-H+6HCOONa]^+^ of Pro | B | 7.8 | 567.95879 | 6.8E+05±3.2E+05 | 1.9E+06±1.1E+06 | 4.1E-03 | 0.4 | -- | LEVEL 1 |
| [M+2Na-H+6HCOONa]^+^ of Val | A | 7.4 | 569.97430 | 5.4E+05±1.3E+05 | 2.1E+05±6.2E+04 | 7.5E-07 | 2.6 | [M+K]^+^ 531.011 [M+Na]^+^ 546.985 [M+H]^+^ 568.967 | LEVEL 1 |
| [4M+5Na-4H+2HCOONa]^+^ of Ala | A | 8.6 | 603.08223 | 8.0E+05±2.5E+05 | 2.3E+05±5.0E+04 | 5.9E-07 | 3.4 | [M+K+NaCOOH]^+^ 496.132 [M+Na+HCOOH]^+^ 534.088 | LEVEL 1 |
| [7M+9Na-7H]2^+^ of Gln | A | 9.3 | 611.16747 | 1.9E+06±3.0E+05 | 3.8E+05±9.5E+04 | 8.2E-13 | 5.1 | [M+Na]^+^ 588.174 [M+H]^+^ 610.159 | LEVEL 1 |
| M+1 13C of [7M+9Na-7H]^2+^ of Gln | A | 9.3 | 611.66900 | 8.5E+05±1.1E+05 | 1.7E+05±5.3E+04 | 4.7E-14 | 5.1 |  | LEVEL 1 |
| [M+2Na-H+7HCOONa]^+^ of Val | A | 7.4 | 637.96179 | 3.7E+05±9.2E+04 | 1.2E+05±4.2E+04 | 1.5E-07 | 3.1 | [M+K+NaCOOH]^+^ 531.011 [M+Na+NaCOOH]^+^ 546.985 [M+Na+HCOOH]^+^ 568.967 | LEVEL 1 |
| [5M+6Na-5H+HCOONa]^+^ of Ala | A | 8.6 | 646.12481 | 5.2E+05±1.1E+05 | 6.5E+04±3.5E+04 | 5.2E-11 | 7.9 | [M+K]^+^ 607.161 [M+Na]^+^ 623.135 [M+H]^+^ 645.117 | LEVEL 1 |
| [4M+5Na-4H]^+^ of Gln | A | 9.3 | 695.19309 | 4.7E+06±7.7E+05 | 1.1E+06±3.1E+05 | 1.2E-11 | 4.2 | -- | LEVEL 1 |
| unidentified | unidentified | 11.0 | 198.71078 | 1.7E+06±2.4E+05 | 2.1E+06±2.0E+05 | 3.0E-03 | 0.8 | -- | LEVEL 5 |
| unidentified | unidentified | 6.0 | 246.09465 | 2.7E+05±1.8E+05 | 1.1E+05±4.9E+04 | 2.2E-02 | 2.6 | -- | LEVEL 5 |
| unidentified | unidentified | 10.5 | 246.18092 | 2.7E+05±5.6E+04 | 3.6E+05±7.4E+04 | 7.5E-03 | 0.7 | -- | LEVEL 5 |
| unidentified | unidentified | 1.3 | 251.10748 | 3.1E+05±1.2E+05 | 8.9E+05±3.9E+05 | 3.0E-04 | 0.3 | -- | LEVEL 5 |
| unidentified | unidentified | 9.1 | 299.02030 | 4.5E+05±7.1E+04 | 2.0E+05±3.5E+04 | 2.9E-09 | 2.2 | -- | LEVEL 5 |
| unidentified | unidentified | 5.9 | 301.93834 | 2.7E+05±6.1E+04 | 5.7E+05±1.1E+05 | 7.3E-07 | 0.5 | -- | LEVEL 5 |
| unidentified | unidentified | 10.4 | 319.99889 | 1.0E+06±2.7E+05 | 2.9E+05±7.9E+04 | 5.1E-08 | 3.6 | [M+H]^+^ 318.99 | LEVEL 5 |
| unidentified | unidentified | 9.2 | 391.95256 | 5.7E+05±1.5E+05 | 3.9E+05±1.6E+05 | 4.1E-02 | 1.5 | [M+H-HCOOH]^+^ 436.951 | LEVEL 5 |
| unidentified | unidentified | 9.4 | 454.59945 | 6.2E+05±1.7E+05 | 1.5E+05±6.9E+04 | 1.2E-07 | 4.1 | -- | LEVEL 5 |
| unidentified | unidentified | 9.3 | 511.16903 | 1.7E+06±2.9E+05 | 5.5E+05±1.7E+05 | 4.1E-10 | 3.2 | -- | LEVEL 5 |
| unidentified | unidentified | 9.3 | 679.21881 | 5.7E+05±1.5E+05 | 9.6E+04±3.4E+04 | 6.0E-09 | 5.9 | [2M+K]^+^ 320.128 | LEVEL 5 |

**Supplemental Table S5. Summary of the biomarker discovery results (FDR = 5%) obtained by comparing the metabolomic profiles of plasma samples mimicking those collected from diseased subjects (n = 6) and non-diseased subjects (n = 6). All differential features observed in this analysis were found in the analysis with the sample size n = 12 for each group (see Table 2 in the main manuscript for details).**

| snthresh (signal-to-noise ratio threshold) for metabolomic feature extraction | Total number of metabolomic features^a^  (M) | Number of differential metabolomic features, i.e., putative biomarkers  (PB) | Number of true positives i.e., true biomarkers identified as putative biomarkers  (TP) | Number of false negatives  (FN = 22 – TP) | Number of false positives, i.e., putative biomarkers which were not the true biomarkers  (FP = PB − TP) | Percentage of metabolomic features which were false positives  (FP / M) | False Negative Rate for the true biomarkers  (FNR = FN / 22) | Actual False Discovery Rate, i.e., False Positive Rate for the putative biomarkers  (FPR = FP / PB) | |
| --- | --- | --- | --- | --- | --- | --- | --- | --- | --- |
| 20 | 377 | 58 | 21 | 1 | 37 | 9.8% | 4.5% | 63.8% | |
| 10 | 546 | 73 | 21 | 1 | 54 | 9.9% | 4.5% | 72.0% | |
| 5 | 758 | 143 | 21 | 1 | 122 | 16.1% | 4.5% | 85.3% | |
| ^a^ The metabolomic features were cleaned up using CAMERA and MS-FLO. Features absent in 80% of the samples were discarded. Then features corresponding to the features with CV >30% in the QC samples were discarded. Moreover, features contributed by the impurities in the metabolomic standards were tracked and excluded from the calculations. | | | | | | | | |  |

**Supplemental Table S6. Summary of the biomarker discovery results (FDR = 5%) obtained by comparing the metabolomic profiles of two groups of negative control plasma samples (n = 12 for each group).**

| ***snthresh* (signal-to-noise ratio threshold) for metabolomic feature extraction** | **Total number of metabolomic features** | **Number of differential metabolomic features, i.e., putative biomarkers** |
| --- | --- | --- |
| 20 | 397 | 0 |
| 10 | 566 | 0 |
| 5 | 751 | 0 |

**Supplemental Table S7. Summary of the false positive biomarkers which were related to the spiked standard metabolites and were also observed as metabolomic features in the LC-HRMS profiles of two pure metabolomic standard mixtures, Set A and Set B.**

| **Metabolomic Standards** | **Classification** | **Number of false positive biomarkers** |
| --- | --- | --- |
| Set A only | In source fragmentation products | 17 |
|  | Adducts | 28 |
|  | Isotopes | 3 |
|  | In-source complex | 3 |
| Set B only | In source fragmentation products | 9 |
|  | Adducts | 21 |
|  | Isotopes | 2 |
|  | complex | 5 |
| Both Set A and Set B | In source fragmentation products | 0 |
|  | Adducts | 0 |
|  | Isotopes | 0 |
|  | In-source complex | 0 |
|  | **Total** | **88** |


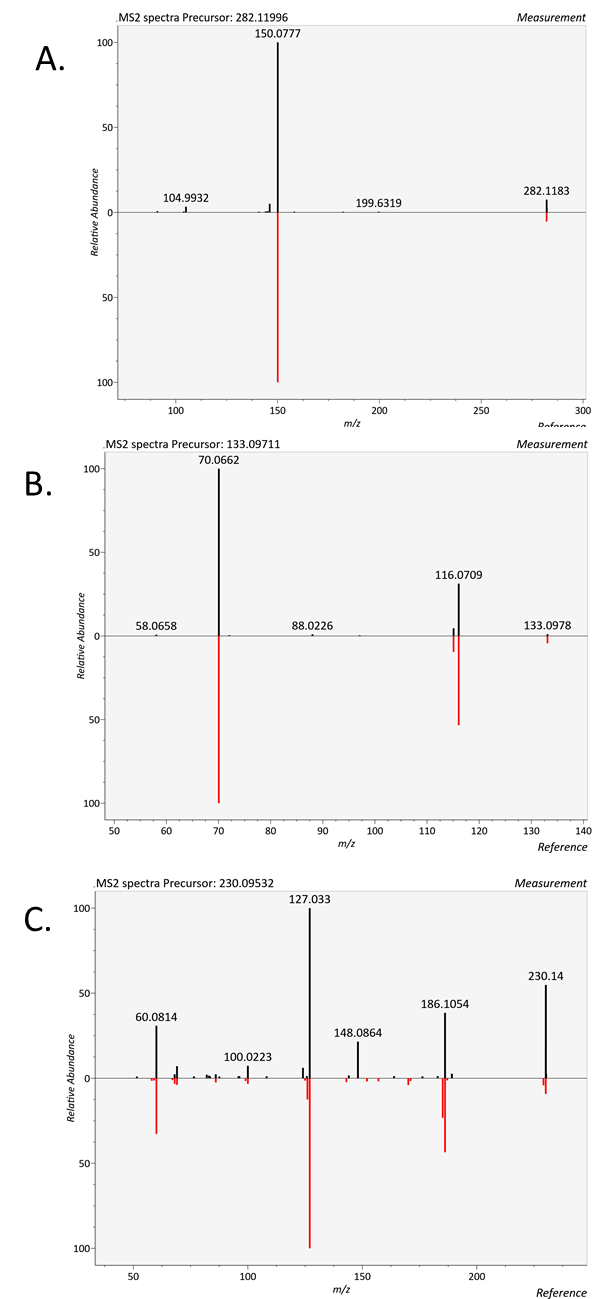


Supplemental Figure S1. Representative MS/MS spectra of 3 false positive biomarkers. 1-Methyladenosine (A), ornithine (B), and ergothioneine (C) were identified to be irrelevant metabolites. In each graph, the upper spectrum (black) is an observed MS/MS spectrum of a false positive biomarker; the lower spectrum (red) is a matched reference MS/MS spectrum of a known metabolite from the MassBank Spectrum Library.

**Supplemental References**

1. Poisson LM, Suhail H, Singh J, et al. Untargeted plasma metabolomics identifies endogenous metabolite with drug-like properties in chronic animal model of multiple sclerosis. J Biol Chem. 2015; 290: 30697–712.

2. Fu Y, Zhang Y, Zhou Z, et al. Screening and determination of potential risk substances based on liquid chromatography–high-resolution mass spectrometry. Anal Chem. 2018; 90: 8454–61.

3. Bruce SJ, Jonsson P, Antti H, et al. Evaluation of a protocol for metabolic profiling studies on human blood plasma by combined ultra-performance liquid chromatography/mass spectrometry: From extraction to data analysis. Analytical Biochemistry. 2008; 372: 237–49.

4. Bruce SJ, Tavazzi I, Parisod V, Rezzi S, Kochhar S, Guy PA. Investigation of human blood plasma sample preparation for performing metabolomics using ultrahigh performance liquid chromatography/mass spectrometry. Anal Chem. 2009; 81: 3285–96.

5. Kouassi Nzoughet J, Bocca C, Simard G, et al. A nontargeted UHPLC-HRMS metabolomics pipeline for metabolite identification: application to cardiac remote ischemic preconditioning. Anal Chem. 2017; 89: 2138–46.

6. Tautenhahn R, Patti GJ, Rinehart D, Siuzdak G. XCMS Online: a web-based platform to process untargeted metabolomic data. Anal Chem. 2012; 84: 5035–9.

7. Holman JD, Tabb DL, Mallick P. Employing ProteoWizard to convert raw mass spectrometry data. Curr Protoc Bioinformatics. 2014; 46: 13.24.1-9.

8. Wu Q, Lai X, Zhao H, et al. A metabolomics approach for predicting the response to intravenous iron therapy in peritoneal dialysis patients with anemia. RSC Advances. 2017; 7: 1915–22.

9. Contrepois K, Jiang L, Snyder M. Optimized analytical procedures for the untargeted metabolomic profiling of human urine and plasma by combining hydrophilic interaction (HILIC) and reverse-phase liquid chromatography (RPLC)–mass spectrometry. Mol Cell Proteomics. 2015; 14: 1684–95.

10. Polpitiya AD, Qian W-J, Jaitly N, et al. DAnTE: a statistical tool for quantitative analysis of -omics data. Bioinformatics. 2008; 24: 1556–8.

11. Li D, Heiling S, Baldwin IT, Gaquerel E. Illuminating a plant’s tissue-specific metabolic diversity using computational metabolomics and information theory. PNAS. 2016; 113: E7610–8.

12. Zhao H, Li H, Chung ACK, et al. Large-scale longitudinal metabolomics study reveals different trimester-specific alterations of metabolites in relation to gestational diabetes mellitus. J Proteome Res. 2019; 18: 292–300.

13. Thévenot EA, Roux A, Xu Y, Ezan E, Junot C. Analysis of the human adult urinary metabolome variations with age, body mass index, and gender by implementing a comprehensive workflow for univariate and OPLS statistical analyses. J Proteome Res. 2015; 14: 3322–35.

14. DeFelice BC, Mehta SS, Samra S, et al. Mass spectral feature list optimizer (MS-FLO): A tool to minimize false positive peak reports in untargeted liquid chromatography–mass spectroscopy (LC-MS) data processing. Anal Chem. 2017; 89: 3250–5.

15. Tsugawa H, Cajka T, Kind T, et al. MS-DIAL: data-independent MS/MS deconvolution for comprehensive metabolome analysis. Nature Methods. 2015; 12: 523–6.

16. Lai Z, Tsugawa H, Wohlgemuth G, et al. Identifying metabolites by integrating metabolome databases with mass spectrometry cheminformatics. Nature Methods. 2018; 15: 53–6.

17. Cynober LA. Plasma amino acid levels with a note on membrane transport: characteristics, regulation, and metabolic significance. Nutrition. 2002; 18: 761–6.

18. Bjerkenstedt L, Edman G, Hagenfeldt L, Sedvall G, Wiesel FA. Plasma amino acids in relation to cerebrospinal fluid monoamine metabolites in schizophrenic patients and healthy controls. Br J Psychiatry. 1985; 147: 276–82.

19. Plasma amino acids: MedlinePlus Medical Encyclopedia. Available at: https://medlineplus.gov/ency/article/003361.htm

20. Jäger R, Harris RC, Purpura M, Francaux M. Comparison of new forms of creatine in raising plasma creatine levels. J Int Soc Sports Nutr. 2007; 4: 17.

21. Salomons GS, van Dooren SJ, Verhoeven NM, et al. X-linked creatine-transporter gene (SLC6A8) defect: a new creatine-deficiency syndrome. Am J Hum Genet. 2001; 68: 1497–500.

22. Stegink LD, Filer LJ, Brummel MC, et al. Plasma amino acid concentrations and amino acid ratios in normal adults and adults heterozygous for phenylketonuria ingesting a hamburger and milk shake meal. Am J Clin Nutr. 1991; 53: 670–5.

23. Zordoky BN, Sung MM, Ezekowitz J, et al. Metabolomic fingerprint of heart failure with preserved ejection fraction. PLoS ONE. 2015; 10: e0124844.

24. Stegink LD, Filer LJ, Bell EF, Ziegler EE. Plasma amino acid concentrations in normal adults administered aspartame in capsules or solution: lack of bioequivalence. Metab Clin Exp. 1987; 36: 507–12.

25. Org E, Blum Y, Kasela S, et al. Relationships between gut microbiota, plasma metabolites, and metabolic syndrome traits in the METSIM cohort. Genome Biology. 2017; 18: 70.

26. Zhao Q, Cao Y, Wang Y, et al. Plasma and tissue free amino acid profiles and their concentration correlation in patients with lung cancer. Asia Pac J Clin Nutr. 2014; 23: 429–36.

27. Altamura C, Maes M, Dai J, Meltzer HY. Plasma concentrations of excitatory amino acids, serine, glycine, taurine and histidine in major depression. Eur Neuropsychopharmacol. 1995; 5: 71–5.

28. Psychogios N, Hau DD, Peng J, et al. The human serum metabolome. PLoS One. 2011; 6: e16957.

29. Vielhaber S, Feistner H, Weis J, et al. Primary carnitine deficiency: adult onset lipid storage myopathy with a mild clinical course. Journal of Clinical Neuroscience. 2004; 11: 919–24.

30. Waber LJ, Valle D, Neill C, DiMauro S, Shug A. Carnitine deficiency presenting as familial cardiomyopathy: a treatable defect in carnitine transport. J Pediatr. 1982; 101: 700–5.

31. Vesali RF, Klaude M, Rooyackers O, Wernerman J. Amino acid metabolism in leg muscle after an endotoxin injection in healthy volunteers. Am J Physiol Endocrinol Metab. 2005; 288: E360-364.

32. Caldeira Araújo H, Smit W, Verhoeven NM, et al. Guanidinoacetate methyltransferase deficiency identified in adults and a child with mental retardation. Am J Med Genet A. 2005; 133A: 122–7.

33. Tavazzi B, Lazzarino G, Leone P, et al. Simultaneous high performance liquid chromatographic separation of purines, pyrimidines, N-acetylated amino acids, and dicarboxylic acids for the chemical diagnosis of inborn errors of metabolism. Clin Biochem. 2005; 38: 997–1008.

34. Toyoshima K, Nakamura M, Adachi Y, et al. Increased plasma proline concentrations are associated with sarcopenia in the elderly. PLoS ONE. 2017; 12: e0185206.

35. Koike S, Bundo M, Iwamoto K, et al. A snapshot of plasma metabolites in first-episode schizophrenia: a capillary electrophoresis time-of-flight mass spectrometry study. Transl Psychiatry. 2014; 4: e379.

36. Laryea MD, Steinhagen F, Pawliczek S, et al. Simple method for the routine determination of betaine and N,N-dimethylglycine in blood and urine. Clin Chem. 1998; 44: 1937-41.

37. Kikuchi T, Orita Y, Ando A, et al. Liquid-chromatographic determination of guanidino compounds in plasma and erythrocyte of normal persons and uremic patients. Clin Chem. 1981; 27: 1899–902.

38. Gatti R, Gioia MG. Liquid chromatographic analysis of guanidino compounds using furoin as a new fluorogenic reagent. J Pharm Biomed Anal. 2008; 48: 754–9.

39. Trabado S, Al-Salameh A, Croixmarie V, et al. The human plasma-metabolome: Reference values in 800 French healthy volunteers; impact of cholesterol, gender and age. PLoS ONE. 2017; 12: e0173615.

40. Schmidt JA, Rinaldi S, Scalbert A, et al. Plasma concentrations and intakes of amino acids in male meat-eaters, fish-eaters, vegetarians and vegans: a cross-sectional analysis in the EPIC-Oxford cohort. Eur J Clin Nutr. 2016; 70: 306–12.

41. Kamoun P, Richard V, Rabier D, Saudubray JM. Plasma lysine concentration and availability of 2-ketoglutarate in liver mitochondria. J Inherit Metab Dis. 2002; 25: 1–6.

42. Matsuda M, Asano Y. Determination of plasma and serum l-lysine using l-lysine ε-oxidase from Marinomonas mediterranea NBRC 103028T. Analytical Biochemistry. 2010; 406: 19–23.

43. Fadel FI, Elshamaa MF, Essam RG, et al. Some amino acids levels: glutamine, glutamate, and homocysteine, in plasma of children with chronic kidney disease. Int J Biomed Sci. 2014; 10: 36–42.

44. Lepage N, McDonald N, Dallaire L, Lambert M. Age-specific distribution of plasma amino acid concentrations in a healthy pediatric population. Clin Chem. 1997; 43: 2397–402.

45. Kock R, Delvoux B, Sigmund M, Greiling H. A comparative study of the concentrations of hypoxanthine, xanthine, uric acid and allantoin in the peripheral blood of normals and patients with acute myocardial infarction and other ischaemic diseases. Eur J Clin Chem Clin Biochem. 1994; 32: 837–42.

46. Kaya M, Moriwaki Y, Ka T, et al. Plasma concentrations and urinary excretion of purine bases (uric acid, hypoxanthine, and xanthine) and oxypurinol after rigorous exercise. Metabolism. 2006; 55: 103–7.

47. Mayers JR, Wu C, Clish CB, et al. Elevation of circulating branched-chain amino acids is an early event in human pancreatic adenocarcinoma development. Nature Medicine. 2014; 20: 1193–8.

48. Mochel F, Benaich S, Rabier D, Durr A. Validation of plasma branched chain amino acids as biomarkers in huntington disease. Arch Neurol. 2011; 68: 264–71.

49. Birech Z, Mwangi PW, Bukachi F, Mandela KM. Application of Raman spectroscopy in type 2 diabetes screening in blood using leucine and isoleucine amino-acids as biomarkers and in comparative anti-diabetic drugs efficacy studies. PLoS ONE. 2017; 12: e0185130.

50. Ribes A, Pajares S, Arias Á, García-Villoria J. Creatine as biomarker. In: General Methods in Biomarker Research and their Applications. Dordrecht: Springer Netherlands; 2015: 333–61.

51. Puleo PR, Guadagno PA, Roberts R, et al. Early diagnosis of acute myocardial infarction based on assay for subforms of creatine kinase-MB. Circulation. 1990; 82: 759–64.

52. Hisamatsu T, Okamoto S, Hashimoto M, et al. Novel, objective, multivariate biomarkers composed of plasma amino acid profiles for the diagnosis and assessment of inflammatory bowel disease. PLoS One. 2012; 7: e31131

53. Lee H-O, Uzzo RG, Kister D, Kruger WD. Combination of serum histidine and plasma tryptophan as a potential biomarker to detect clear cell renal cell carcinoma. J Transl Med. 2017; 15: 72.

54. Zhai G, Wang-Sattler R, Hart DJ, et al. Serum branched-chain amino acid to histidine ratio: a novel metabolomic biomarker of knee osteoarthritis. Ann Rheum Dis. 2010; 69: 1227–31.

55. Wang TJ, Larson MG, Vasan RS, et al. Metabolite profiles and the risk of developing diabetes. Nature Medicine. 2011; 17: 448–53.

56. Steventon GB, Mitchell SC. Phenylalanine hydroxylase: A biomarker of disease susceptibility in Parkinson’s disease and Amyotrophic lateral sclerosis. Med Hypotheses. 2018; 118: 29–33.

57. Neumann S, Welling H, Thuere S. Evaluation of serum L-phenylalanine concentration as indicator of liver disease in dogs: a pilot study. J Am Anim Hosp Assoc. 2007; 43: 193–200.

58. Wiggins T, Kumar S, Markar SR, Antonowicz S, Hanna GB. Tyrosine, phenylalanine, and tryptophan in gastroesophageal malignancy: a systematic review. Cancer Epidemiol Biomarkers Prev. 2015; 24: 32–8.

59. Expert Committee on the Diagnosis and Classification of Diabetes Mellitus. Report of the expert committee on the diagnosis and classification of diabetes mellitus. Diabetes Care. 2003; 26 Suppl 1: S5-20.

60. Hartman M-L, Goodson JM, Shi P, et al. Unhealthy Phenotype as Indicated by Salivary Biomarkers: Glucose, Insulin, VEGF-A, and IL-12p70 in Obese Kuwaiti Adolescents. J Obes. 2016; 2016: 6860240.

61. Park WG, Wu M, Bowen R, et al. Metabolomic-derived novel cyst fluid biomarkers for pancreatic cysts: glucose and kynurenine. Gastrointest Endosc. 2013; 78: 295-302.e2.

62. Barderas MG, Laborde CM, Posada M, et al. Metabolomic profiling for identification of novel potential biomarkers in cardiovascular diseases. J Biomed Biotechnol. 2011; 2011: 790132.

63. Guma M, Tiziani S, Firestein GS. Metabolomics in rheumatic diseases: desperately seeking biomarkers. Nat Rev Rheumatol. 2016; 12: 269–81.

64. Nishiumi S, Kobayashi T, Ikeda A, et al. A novel serum metabolomics-based diagnostic approach for colorectal cancer. PLoS ONE. 2012; 7: e40459.

65. Shen X, Deng C, Wang B, Dong L. Quantification of trimethylsilyl derivatives of amino acid disease biomarkers in neonatal blood samples by gas chromatography-mass spectrometry. Anal Bioanal Chem. 2006; 384: 931–8.

66. Deng C, Deng Y. Diagnosis of maple syrup urine disease by determination of L-valine, L-isoleucine, L-leucine and L-phenylalanine in neonatal blood spots by gas chromatography-mass spectrometry. J Chromatogr B Analyt Technol Biomed Life Sci. 2003; 792: 261–8.

67. Arsik I, Frediani JK, Frezza D, et al. Alanine aminotransferase as a monitoring biomarker in children with nonalcoholic fatty liver disease: a secondary analysis using TONIC trial data. Children (Basel). 2018 ;5: 64.

68. Martin-Rodriguez JL, Gonzalez-Cantero J, Gonzalez-Cantero A, Arrebola JP, Gonzalez-Calvin JL. Diagnostic accuracy of serum alanine aminotransferase as biomarker for nonalcoholic fatty liver disease and insulin resistance in healthy subjects, using 3T MR spectroscopy. Medicine (Baltimore). 2017; 96: e6770.

69. Buzkova J, Nikkanen J, Ahola S, et al. Metabolomes of mitochondrial diseases and inclusion body myositis patients: treatment targets and biomarkers. EMBO Mol Med. 2018; 10.

70. Shaham O, Slate NG, Goldberger O, et al. A plasma signature of human mitochondrial disease revealed through metabolic profiling of spent media from cultured muscle cells. Proc Natl Acad Sci USA. 2010; 107: 1571–5.

71. González-Domínguez R, García-Barrera T, Gómez-Ariza JL. Application of a novel metabolomic approach based on atmospheric pressure photoionization mass spectrometry using flow injection analysis for the study of Alzheimer’s disease. Talanta. 2015; 131: 480–9.

72. Gruber B, Kłaczkow G, Jaworska M, et al. Huntington’ disease--imbalance of amino acid levels in plasma of patients and mutation carriers. Ann Agric Environ Med. 2013; 20: 779–83.

73. Madeira C, Lourenco MV, Vargas-Lopes C, et al. d-serine levels in Alzheimer’s disease: implications for novel biomarker development. Transl Psychiatry. 2015; 5: e561.

74. Nagata Y, Hirayama A, Ikeda S, et al. Comparative analysis of cerebrospinal fluid metabolites in Alzheimer’s disease and idiopathic normal pressure hydrocephalus in a Japanese cohort. Biomark Res. 2018; 6: 5.

75. Kang J, Lu J, Zhang X. Metabolomics-based promising candidate biomarkers and pathways in Alzheimer’s disease. Pharmazie. 2015; 70: 277–82.

76. Liu P, Li R, Antonov AA, et al. Discovery of metabolite biomarkers for acute ischemic stroke progression. J Proteome Res. 2017; 16: 773–9.

77. Ahmed HH. Modulatory effects of vitamin E, Acetyl-L-carnitine and α-lipoic acid on new potential biomarkers for Alzheimer’s disease in rat model. Exp Toxicol Pathol. 2012; 64: 549–56.

78. Frye RE, Melnyk S, Macfabe DF. Unique acyl-carnitine profiles are potential biomarkers for acquired mitochondrial disease in autism spectrum disorder. Transl Psychiatry. 2013; 3: e220.

79. Strand E, Pedersen ER, Svingen GFT, et al. Serum acylcarnitines and risk of cardiovascular death and acute myocardial infarction in patients with stable angina pectoris. J Am Heart Assoc. 2017; 6.

80. Smith AM, King JJ, West PR, et al. Amino acid dysregulation metabotypes: potential biomarkers for diagnosis and individualized treatment for subtypes of autism spectrum disorder. Biol Psychiatry. 2019; 85: 345–54.

81. Santarpia L, Catanzano F, Ruoppolo M, et al. Citrulline blood levels as indicators of residual intestinal absorption in patients with short bowel syndrome. Ann Nutr Metab. 2008; 53: 137–42.

82. Rhee SY, Jung ES, Park HM, et al. Plasma glutamine and glutamic acid are potential biomarkers for predicting diabetic retinopathy. Metabolomics. 2018; 14: 89.

83. Yan J-K, Zhou K-J, Huang J-H, et al. Urinary glutamine/glutamate ratio as a potential biomarker of pediatric chronic intestinal pseudo-obstruction. Orphanet J Rare Dis. 2017; 12: 62.

84. Morris CR, Suh JH, Hagar W, et al. Erythrocyte glutamine depletion, altered redox environment, and pulmonary hypertension in sickle cell disease. Blood. 2008; 111: 402–10.

85. van Eijk RPA, Eijkemans MJC, Ferguson TA, Nikolakopoulos S, Veldink JH, van den Berg LH. Monitoring disease progression with plasma creatinine in amyotrophic lateral sclerosis clinical trials. J Neurol Neurosurg Psychiatry. 2018; 89: 156–61.

86. Lopez-Giacoman S, Madero M. Biomarkers in chronic kidney disease, from kidney function to kidney damage. World J Nephrol. 2015; 4: 57–73.

87. Fan Y, Zhou X, Xia T-S, et al. Human plasma metabolomics for identifying differential metabolites and predicting molecular subtypes of breast cancer. Oncotarget. 2016; 7: 9925–38.

88. Viswan A, Singh C, Rai RK, Azim A, Sinha N, Baronia AK. Metabolomics based predictive biomarker model of ARDS: A systemic measure of clinical hypoxemia. PLoS ONE. 2017; 12: e0187545.

89. Xie G, Lu L, Qiu Y, et al. Plasma metabolite biomarkers for the detection of pancreatic cancer. J Proteome Res. 2015; 14: 1195–202.

90. Liu X, Gao J, Chen J, et al. Identification of metabolic biomarkers in patients with type 2 diabetic coronary heart diseases based on metabolomic approach. Sci Rep. 2016; 6: 30785.

91. Shah SH, Kraus WE, Newgard CB. Metabolomic profiling for the identification of novel biomarkers and mechanisms related to common cardiovascular diseases: form and function. Circulation. 2012; 126: 1110–20.

92. Falegan OS, Ball MW, Shaykhutdinov RA, et al. Urine and Serum Metabolomics Analyses May Distinguish between Stages of Renal Cell Carcinoma. Metabolites. 2017; 7.

93. Ubhi BK, Riley JH, Shaw PA, et al. Metabolic profiling detects biomarkers of protein degradation in COPD patients. Eur Respir J. 2012; 40: 345–55.

94. Stoessel D, Schulte C, Teixeira Dos Santos MC, et al. Promising metabolite profiles in the plasma and CSF of early clinical Parkinson’s disease. Front Aging Neurosci. 2018; 10: 51.

95. Finkelman BS, Putt M, Wang T, et al. Arginine-nitric oxide metabolites and cardiac dysfunction in patients with breast cancer. J Am Coll Cardiol. 2017; 70: 152–62.

96. Chen H, Cao G, Chen D-Q, et al. Metabolomics insights into activated redox signaling and lipid metabolism dysfunction in chronic kidney disease progression. Redox Biol. 2016; 10: 168–78.

97. Wang X, Zhang A, Han Y, et al. Urine metabolomics analysis for biomarker discovery and detection of jaundice syndrome in patients with liver disease. Mol Cell Proteomics. 2012; 11: 370–80.

98. Yu J, Kong L, Zhang A, et al. High-throughput metabolomics for discovering potential metabolite biomarkers and metabolic mechanism from the APPswe/PS1dE9 transgenic model of Alzheimer’s disease. J Proteome Res. 2017; 16: 3219–28.

99. Viswan A, Singh C, Rai RK, Azim A, Sinha N, Baronia AK. Metabolomics based predictive biomarker model of ARDS: A systemic measure of clinical hypoxemia. PLoS ONE. 2017; 12: e0187545.

100. Chen T, Xie G, Wang X, et al. Serum and urine metabolite profiling reveals potential biomarkers of human hepatocellular carcinoma. Mol Cell Proteomics. 2011; 10: M110.004945.

101. Pegalajar-Jurado A, Fitzgerald BL, Islam MN, et al. Identification of urine metabolites as biomarkers of early Lyme disease. Sci Rep. 2018; 8: 12204.

102. Liu X, Zheng P, Zhao X, et al. Discovery and validation of plasma biomarkers for major depressive disorder classification based on liquid chromatography-mass spectrometry. J Proteome Res. 2015; 14: 2322–30.

103. Saudubray J-M, Rabier D. Biomarkers identified in inborn errors for lysine, arginine, and ornithine. J Nutr. 2007; 137: 1669S–72S.

104. Stanislovaitienė D, Žaliūnienė D, Steponavičiūtė R, Žemaitienė R, Gustienė O, Žaliūnas R. N-carboxymethyllysine as a biomarker for coronary artery disease and age-related macular degeneration. Medicina (Kaunas). 2016; 52: 99–103.

105. Zhang X, Lai Y, McCance DR, et al. Evaluation of N (epsilon)-(3-formyl-3,4-dehydropiperidino)lysine as a novel biomarker for the severity of diabetic retinopathy. Diabetologia. 2008; 51: 1723–30.

106. Yan J-K, Zhou K-J, Huang J-H, et al. Urinary glutamine/glutamate ratio as a potential biomarker of pediatric chronic intestinal pseudo-obstruction. Orphanet J Rare Dis. 2017; 12: 62.

107. Institute of Medicine. 2011. Glutamate-related biomarkers in drug development for disorders of the nervous system: workshop summary. Washington, DC: The National Academies Press.

108. Lorenzi PL, Llamas J, Gunsior M, et al. Asparagine synthetase is a predictive biomarker of L-asparaginase activity in ovarian cancer cell lines. Mol Cancer Ther. 2008; 7: 3123–8.

109. Jiang S, Hinchliffe TE, Wu T. Biomarkers of an autoimmune skin disease--psoriasis. Genomics Proteomics Bioinformatics. 2015; 13: 224–33.

110. Lee JS, Wang RX, Alexeev EE, et al. Hypoxanthine is a checkpoint stress metabolite in colonic epithelial energy modulation and barrier function. J Biol Chem. 2018; 293: 6039–51.

111. Farthing DE, Farthing CA, Xi L. Inosine and hypoxanthine as novel biomarkers for cardiac ischemia: from bench to point-of-care. Exp Biol Med (Maywood). 2015; 240: 821–31.

112. Weiner J, Parida SK, Maertzdorf J, et al. Biomarkers of inflammation, immunosuppression and stress with active disease are revealed by metabolomic profiling of tuberculosis patients. PLoS ONE. 2012; 7: e40221.

113. Yoo BC, Kong S-Y, Jang S-G, et al. Identification of hypoxanthine as a urine marker for non-Hodgkin lymphoma by low-mass-ion profiling. BMC Cancer. 2010; 10: 55.

114. Lewis GD, Wei R, Liu E, et al. Metabolite profiling of blood from individuals undergoing planned myocardial infarction reveals early markers of myocardial injury. J Clin Invest. 2008; 118: 3503–12.

115. Guo L, Tan G, Liu P, et al. Three plasma metabolite signatures for diagnosing high altitude pulmonary edema. Scientific Reports. 2015; 5: 15126.

116. Hung C-S, Li H-Y, Kuo C-H, et al. Fasting but not changes of plasma metabolome during oral glucose tolerance tests improves the diagnosis of severe coronary arterial stenosis. Clinical Endocrinology. 2015; 83: 483–9.

117. Yang Z, Zhao A, Li Z, et al. Metabolomics reveals positive acceleration(+Gz)-induced metabolic perturbations and the protective effect of Ginkgo biloba extract in a rat model based on ultra high-performance liquid chromatography coupled with quadrupole time-of-flight mass spectrometry. Journal of Pharmaceutical and Biomedical Analysis. 2016; 125: 77–84.

118. Gao S, Yang R, Peng Z, et al. Metabolomics analysis for hydroxy-L-proline-induced calcium oxalate nephrolithiasis in rats based on ultra-high performance liquid chromatography quadrupole time-of-flight mass spectrometry. Scientific Reports. 2016; 6: 30142.

119. Liu Y, Hong Z, Tan G, et al. NMR and LC/MS-based global metabolomics to identify serum biomarkers differentiating hepatocellular carcinoma from liver cirrhosis. International Journal of Cancer. 2014; 135: 658–68.

120. Liu J, Liu J, Zhan G, et al. UPLC‑QTOFMS‑based metabolomic analysis of the serum of hypoxic preconditioning mice. Molecular Medicine Reports. 2017; 16: 6828–36.

121. Liao W-T, Liu B, Chen J, et al. Metabolite modulation in human plasma in the early phase of acclimatization to hypobaric hypoxia. Scientific Reports. 2016; 6: 22589.

122. Kuligowski J, Pérez-Guaita D, Sánchez-Illana Á, et al. Analysis of multi-source metabolomic data using joint and individual variation explained (JIVE). Analyst. 2015; 140: 4521–9.

123. Liu C, Liu B, Liu L, et al. Arachidonic acid metabolism pathway is not only dominant in metabolic modulation but associated with phenotypic variation after acute hypoxia exposure. Front Physiol. 2018: 00236.

124. Nielsen KL, Telving R, Andreasen MF, Hasselstrøm JB, Johannsen M. A metabolomics study of retrospective forensic data from whole blood samples of humans exposed to 3,4-methylenedioxymethamphetamine: a new approach for identifying drug metabolites and changes in metabolism related to drug consumption. J Proteome Res. 2016; 15: 619–27.

125. Alexandre-Gouabau M-C, Courant F, Moyon T, et al. Maternal and cord blood LC-HRMS metabolomics reveal alterations in energy and polyamine metabolism, and oxidative stress in very-low birth weight infants. J Proteome Res. 2013; 12: 2764–78.

126. Xue J, Lai Y, Chi L, et al. Serum metabolomics reveals that gut microbiome perturbation mediates metabolic disruption induced by arsenic exposure in mice. J Proteome Res. 2019 1; 18: 1006-18

127. Lawler NG, Abbiss CR, Gummer JPA, et al. Characterizing the plasma metabolome during 14 days of live-high, train-low simulated altitude: A metabolomic approach. Experimental Physiology. 2019; 104: 81–92.

128. Fan L, Yin M, Ke C, et al. Use of plasma metabolomics to identify diagnostic biomarkers for early stage epithelial ovarian cancer. J Cancer. 2016; 7: 1265–72.

129. Wang H, Liu Z, Wang S, et al. UHPLC-Q-TOF/MS based plasma metabolomics reveals the metabolic perturbations by manganese exposure in rat models. Metallomics. 2017; 9: 192–203.

130. He X, Zheng N, He J, et al. Gut microbiota modulation attenuated the hypolipidemic effect of simvastatin in high-fat/cholesterol-diet fed mice. J Proteome Res. 2017; 16: 1900–10.

131. Yang L, Yang X, Kong X, et al. Covariation analysis of serumal and urinary metabolites suggests aberrant glycine and fatty acid metabolism in chronic Hepatitis B. PLOS ONE. 2016; 11: e0156166.

132. Wang H, Liu A, Zhao W, et al. Metabolomics research reveals the mechanism of action of astragalus polysaccharide in rats with digestive system disorders. Molecules. 2018; 23: 3333.
